# GE-IA-NAM: gene–environment interaction analysis via imaging-assisted neural additive model

**DOI:** 10.1093/bioinformatics/btaf481

**Published:** 2025-08-29

**Authors:** Jingmao Li, Yaqing Xu, Shuangge Ma, Kuangnan Fang

**Affiliations:** Department of Biostatistics, Yale School of Public Health, New Haven, CT 06511, United States; School of Public Health, Shanghai Jiao Tong University School of Medicine, Shanghai 200025, China; Department of Biostatistics, Yale School of Public Health, New Haven, CT 06511, United States; Department of Statistics and Data Science, School of Economics, Xiamen University, Xiamen 361005, China

## Abstract

**Motivation:**

Gene–environment (G–E) interaction analysis is crucial in cancer research, offering insights into how genetic and environmental factors jointly influence cancer outcomes. Most existing G–E interaction methods are regression-based, which may lack flexibility to capture complex data patterns. Recent advances have investigated deep neural network-based G–E models. However, these methods may be more vulnerable to information deficiency due to challenges such as limited sample size and high dimensionality. Apart from genetic and environmental data, pathological images have emerged as a widely accessible and informative resource for cancer modeling, presenting its potential to enhance G–E modeling.

**Results:**

We propose the pathological imaging-assisted neural additive model for G–E analysis (GE-IA-NAM). The flexible and interpretable additive network architecture is adopted to account for individualized effects associated with genetic factors, environmental factors, and their interactions. To improve G–E modeling, an assisted-learning strategy is investigated, which adopts a joint analysis to integrate information from pathological images. Simulations and the analysis of lung and skin cancer datasets from The Cancer Genome Atlas demonstrate the competitive performance of the proposed method.

**Availability and implementation:**

Python code implementing the proposed method is available at https://github.com/Mr-maoge/NAM-IA-GE. The data that support the findings in this article are openly available in TCGA (The Cancer Genome Atlas) at https://portal.gdc.cancer.gov/.

## 1 Introduction

The prognosis of cancers is closely associated with the combined effects of genes (and other omics variables), environmental factors (including clinical and demographic variables), and gene-environment (G–E) interactions (Hunter 2005). Extensive statistical investigations have been conducted, aiming to select important variables or build prediction models (Herrera-Luis *et al.* 2024). However, traditional G–E analysis methods are primarily regression-based. While straightforward, these methods may fail to capture the complex, unknown, and nonlinear effects, thereby limiting their applicability.

Recently, deep neural network (DNN)-based biomedical studies have gained significant interest. Compared to linear models, deep learning approaches can be more flexible in capturing complex patterns and dependencies (Kang *et al.* 2022). Various investigations have been made, as detailed in comprehensive surveys (Li *et al.* 2022, Yu *et al.* 2023). Specifically, targeting the inherent high-dimensional challenges in omics data analysis, numerous variable selection and dimension reduction methods for DNN have been explored. For example, Chen *et al.* (2021) added a new “selection layer” before the input layer to facilitate variable selection. Zhu *et al.* (2021) developed the deep learning-based knockoffs inference for variable selection with a false discovery rate control. Perhaps the most relevant literature, Wu *et al.* (2023) developed the DeepGE method, which incorporates all G variables, E variables, and G–E interactions into a fully connected DNN, and developed a penalized estimation to facilitate variable selection while respecting the “main effects, interactions” heredity. Although straightforward, the use of a fully connected architecture may obscure the direct influence of individual variables. Moreover, the method models interactions both explicitly—by including them directly in the input layer—and implicitly—through the network’s nonlinear transformations. This dual mechanism may also reduce explainability. Therefore, it is of interest to explore alternative DNN architectures that offer improved interpretability.

Another challenge in DNN-based G–E analysis is information deficiency. Compared to main-effect-only analysis or regression-based methods, G–E interaction analysis under deep learning is inherently more difficult, due to high dimensionality, increased model complexity, and the “main effects, interactions” variable selection heredity constraint (Hunter 2005). To mitigate such problem, substantial efforts have been focused on developing assisted-learning strategies that incorporate auxiliary information from diverse sources and data types. For example, transfer learning methods (Elkayam *et al.* 2024) aim to address the information deficiency by leveraging data or model information from related tasks. Multimodal analysis (Magazzù *et al.* 2021) integrates information from various modalities, such as different types of omics data, through joint analysis, thereby improving the model performance. Other types of auxiliary information, including pathway structures (Jablonski and Beerenwinkel 2023), prior knowledge from literature (Wang *et al.* 2023), and structural characteristics of G measurements (Wu *et al.* 2020) have also been investigated. Given the intrinsic differences across these auxiliary data types, customized methods are typically required.

Following a similar path as previous literature, we propose using pathological images as auxiliary information. In cancer modeling, pathological images have become an invaluable resource due to their accessibility and cost-effectiveness. These images are routinely generated as part of standard diagnostic and therapeutic workflows in clinical cancer practice (Yuan *et al.* 2012), making them widely available. They provide critical biological insights into tumors, which are essential for evaluating prognosis in cancer research. Furthermore, studies have attempted to integrate imaging and omics data for cancer analysis (Fan *et al.* 2024), or to elucidate the close relationships between these two data modalities (Zhang *et al.* 2020).

In this study, we propose to conduct G–E interaction analysis via the pathological imaging-assisted neural additive model (NAM). The model is built upon the interpretable NAM architecture (Agarwal *et al.* 2021), which imposes an additive structure on neural networks, allowing for the evaluation of each variable’s individual effect. The method has been widely investigated (Utkin *et al.* 2022, Ortega-Fernandez *et al.* 2024) and also adopted in interaction analysis (Yang *et al.* 2021). Furthermore, we introduce an assisted-learning strategy that integrates pathological imaging data to enhance G–E interaction modeling. This joint estimation leverages the inherent relationship between genetic variables and imaging features.

The proposed method may advance existing approaches in several aspects. First, the proposed NAM-based G–E model strikes a balance between model complexity and interoperability. It offers greater flexibility than regression-based G–E analysis methods. When compared to traditional additive models—such as those employing B-spline expansions—NAM may also capture more complex dependencies and enhance model performance (Thielmann *et al.* 2024). Moreover, it eliminates the need to specify the degree, number, or placement of knots, facilitating more convenient end-to-end model estimation (Agarwal *et al.* 2021). Meanwhile, in contrast to DeepGE (Wu *et al.* 2023), the proposed model directly estimates an individualized nonlinear effect for each variable, aligning more closely with regression-based approaches and offering enhanced interpretability. Second, by using penalized estimation, the proposed method can perform variable selection while respecting the desired heredity. Lastly, the proposed method may also contribute to the development of assisted-learning strategies or multimodal analysis. Compared with previous studies integrating imaging and omics data, the proposed strategy adopts a distinct objective and methodology, which is novel and holds potential for further extensions.

## 2 Materials and methods

Denote the dateset with *n* i.i.d. samples as ${\left\{(d_{i},z_{i},x_{i},Y_{i},\delta_{i})\right\}}_{i=1}^{n}$. Here, $d_{i}={\left(d_{i,1},\ldots ,d_{i,q}\right)}^{⊤}$ denotes the *q*-dimensional vector of E variables, $z_{i}={\left(z_{i,1},\ldots ,z_{i,r}\right)}^{⊤}$ denotes the *r*-dimensional vector of G variables, and $x_{i}={\left(x_{i,1},\ldots ,x_{i,p}\right)}^{⊤}$ denotes the *p*-dimensional vector of pathological imaging features. With right censoring, we define $T_{i}$ and $C_{i}$ as the event and censoring time. $Y_{i}=\min(T_{i},C_{i})$ and $\delta_{i}=I(T_{i}\leq C_{i})$ denote the observed survival time and censoring indicator, respectively. Our primary objective is to develop a NAM-based G–E model for survival analysis and implement a joint learning strategy that integrates imaging features to improve model performance.

### 2.1 Neural additive network for G–E analysis

**Figure 1.**
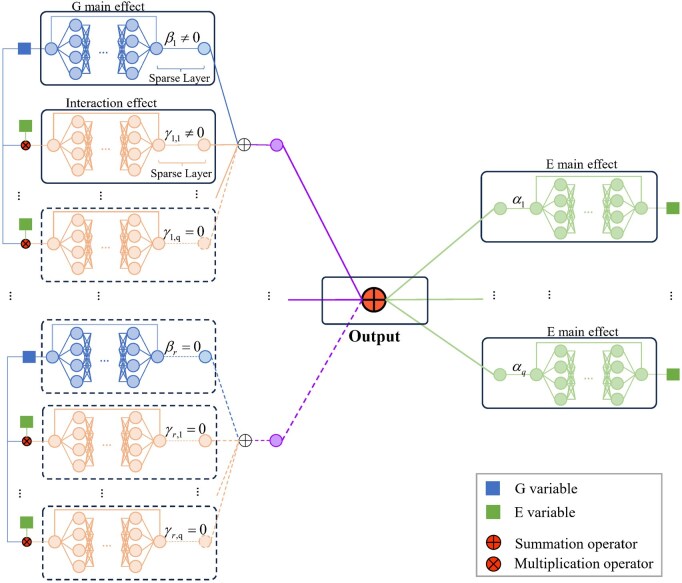
Neural additive network for G–E analysis.

We propose a neural additive network for G–E analysis, as illustrated in Fig. 1. For each G variable, E variable, and G–E interaction, we develop the G sub-net, E sub-net, and interaction sub-net to represent their individualized effects on the output. Each sub-net consists of a residual-connected fully connected network (r-net) and a sparse layer. For r-net, we design a fully connected neural network with a single-variable input for modeling. This network consists of multiple hidden layers that perform affine transformations and introduce nonlinearity through activation functions. Additionally, we adopt a residual connection (He *et al.* 2016) to directly link the input to the output, which explicitly accounts for the linear effect and is helpful in model estimation. Specifically, for input variable x∈R, the formulation g(x) for r-net can be compositely defined as:

(1) $$g(x)=x+g_{L}{}^{\circ}\sigma {}^{\circ}g_{L-1}{}^{\circ}\cdots {}^{\circ}\sigma {}^{\circ}g_{1}(x),$$

where we consider the (L+1)-layer sub-net with layer sizes $p={\left(p_{1},\ldots ,p_{L+1}\right)}^{⊤}$. The dimensions for the input and output layers are $p_{1}=p_{{}_{L+1}}=1$. Function $g_{l}(x)=W_{l}x+v_{l}$ (l=1,…,L) defined with $x\in R^{p_{l}}$ as input, $W_{l}\in R^{p_{l+1}\times p_{l}}$ as the weight matrix, and $v_{l}\in R^{p_{l}}$ as the bias vector. σ(·) is the element-wise nonlinear activation function. We consider rectified linear unit (ReLU) σ(x)=max{x,0} as in literature. For the sparse layer, we perform a one-to-one linear mapping of the input variable, enabling the selection of important G variables and interactions. If the coefficient associated with a variable’s linear mapping is zero, the variable has no effect on the output and is therefore considered as unimportant. Otherwise, it is deemed as important. This approach is widely adopted in the high-dimensional DNN literature (Chen *et al.* 2021, Liu *et al.* 2022).

We model the survival outcome similarly to the Cox model. The conditional hazard function is given by $h_{i}^{\left(1\right)}(t|z_{i},d_{i})=h_{0}^{\left(1\right)}(t)\;\text{exp}[f_{i}^{\left(1\right)}(t)]$ with $h_{0}^{\left(1\right)}(t)$ being the baseline hazard function, and $f_{i}^{\left(1\right)}(t)$ defined as:

(2) $$\begin{array}{l} f_{i}^{\left(1\right)}(t)=\sum_{j=1}^{r}B_{j}(z_{i,j},d_{i})+\sum_{k=1}^{q}\alpha_{k}A_{k}(d_{i,k})\\ =\sum_{j=1}^{r}\beta_{j}B_{j}(z_{i,j})+\sum_{k=1}^{q}\sum_{j=1}^{r}\gamma_{k,j}\Gamma_{k,j}(d_{i,k}z_{i,j})+\sum_{k=1}^{q}\alpha_{k}A_{k}(d_{i,k}). \end{array}$$

Additive terms $\beta_{j}B_{j}(z_{i,j})$ (j=1,…,r), $\alpha_{k}A_{k}(d_{i,k})$ (j=1,…,r, k=1,…,q), and $\gamma_{k,j}\Gamma_{k,j}(d_{i,k}z_{i,j})$ (k=1,…,q) denote the outputs from the G sub-nets, E sub-nets, and interaction sub-nets, with functions $B_{j}(\cdot )$, $A_{k}(\cdot )$, and $\Gamma_{k,j}(\cdot )$ being the corresponding r-net*s* in the sub-nets, and $\beta_{j}$, $\alpha_{k}$, and $\gamma_{k,j}$ being the coefficients in sparse layers. Additionally, for j=1,…,r, we use function $B_{j}(z_{i,j},d_{i})=\beta_{j}B_{j}(z_{i,j})+\sum_{k=1}^{q}\gamma_{k,j}\Gamma_{k,j}(d_{i,k}z_{i,j})$ to represent all the effects corresponding to the *j*th gene. We further denote coefficients vectors $b_{j}^{\left(1\right)}={\left(\beta_{j},\gamma_{1,j},\ldots ,\gamma_{q,j}\right)}^{⊤}$ (j=1,…,r), $\alpha ={\left(\alpha_{1},\ldots ,\alpha_{q}\right)}^{⊤}$, and $\psi^{\left(1\right)}={\left(b_{1}^{(1)⊤},\ldots ,b_{r}^{(1)⊤},\alpha^{⊤}\right)}^{⊤}$.

### 2.2 Pathological imaging-assisted joint learning method

To utilize the information contained in pathological imaging data, we further construct a survival model based on imaging features $x_{i}$. In particular, parallel to the above development, we specify the conditional hazard function based on an imaging data-based interaction model $h_{i}^{\left(2\right)}(t|x_{i},d_{i})=h_{0}^{\left(2\right)}(t)\;\text{exp}[f_{i}^{\left(2\right)}(t)]$, where $h_{0}^{\left(2\right)}(t)$ denotes the corresponding baseline hazard function, and $f_{i}^{\left(2\right)}(t)$ is defined as:

(3) $$\begin{array}{l} f_{i}^{\left(2\right)}(t)=\sum_{j^{\prime }=1}^{p}U_{j^{\prime }}(x_{i,j^{\prime }},d_{i})+\sum_{k=1}^{q}\eta_{k}T_{k}(d_{i,k})\\ =\sum_{j^{\prime }=1}^{p}\theta_{j^{\prime }}U_{j^{\prime }}(x_{i,j^{\prime }})+\sum_{k=1}^{q}\sum_{j^{\prime }=1}^{p}\mu_{k,j^{\prime }}V_{k,j^{\prime }}(d_{i,k}x_{i,j^{\prime }})\\ +\sum_{k=1}^{q}\eta_{k}T_{k}(d_{i,k}). \end{array}$$

Functions $U_{j^{\prime }}(\cdot )$’s, $V_{k,j^{\prime }}(\cdot ,\cdot )$’s, $T_{k}(\cdot )$’s and coefficients $\theta_{j^{\prime }}$’s, $\eta_{k}$’s, $\mu_{k,j^{\prime }}$’s have analogical meanings as those in the G–E model. We also denote function $U_{j^{\prime }}(x_{i,j^{\prime }},d_{i})=\theta_{j^{\prime }}U_{j^{\prime }}(x_{i,j^{\prime }})+\sum_{k=1}^{q}\mu_{k,j^{\prime }}V_{k,j^{\prime }}(d_{i,k},x_{i,j^{\prime }})$ and coefficients vectors $b_{j^{\prime }}^{\left(2\right)}={\left(\theta_{j^{\prime }},\mu_{1,j^{\prime }},\ldots ,\mu_{q,j^{\prime }}\right)}^{⊤}$ ($j^{\prime }=1,\ldots ,p$), $\eta ={\left(\eta_{1},\ldots ,\eta_{q}\right)}^{⊤}$, and $\psi^{\left(2\right)}={\left(b_{1}^{(2)⊤},\ldots ,b_{p}^{(2)⊤},\eta^{⊤}\right)}^{⊤}$. This imaging data-based interacction model is mathematically sensible and can potentially provide some valuable biological insights.

Given the close relationship between G variables and imaging features (Zhang *et al.* 2020), we propose a novel assisted-learning strategy to improve the estimation of G–E model (2) by jointly analyzing both the G–E model and the imaging data-based interaction model (3). The objective function for this joint estimation is

(4) $$\begin{array}{l} Q_{n}(\psi^{\left(1\right)},\Theta^{\left(1\right)},\psi^{\left(2\right)},\Theta^{\left(2\right)})\\ =-L_{n}^{\left(1\right)}(\psi^{\left(1\right)},\Theta^{\left(1\right)})-L_{n}^{\left(2\right)}(\psi^{\left(2\right)},\Theta^{\left(2\right)})\\ +R_{a}^{\left(1\right)}(\psi^{\left(1\right)},\lambda_{1})+R_{a}^{\left(2\right)}(\psi^{\left(2\right)},\lambda_{2})\\ +\lambda_{3}\sum_{j=1}^{r}\sum_{j^{\prime }=1}^{p}\{c_{j,j^{\prime }}\sum_{i=1}^{n}{\left[B_{j}(z_{i,j},d_{i})-U_{j^{\prime }}(x_{i,j^{\prime }},d_{i})\right]}^{2}\}, \end{array}$$

where $\Theta^{\left(1\right)}$ and $\Theta^{\left(2\right)}$ denote all parameters in the sub-networks of the G–E and imaging data-based interaction models. Function $L_{n}^{\left(1\right)}(\psi^{\left(1\right)},\Theta^{\left(1\right)})$ denotes the log partial likelihood for the G–E model, defined as:

$$L_{n}^{\left(1\right)}(\psi^{\left(1\right)},\Theta^{\left(1\right)})=\frac{1}{n}\sum_{i=1}^{n}\delta_{i}\left[f_{i}^{\left(1\right)}(t)-\text{log}\;\left\{\sum_{i^{\prime }\in I_{i}}\;\text{exp}\;\left[f_{i}^{\left(1\right)}(t)\right]\right\}\right],$$

where $I_{i}=\{i^{\prime }:Y_{i^{\prime }}\geq Y_{i}\}$ (i=1,…n) denotes the at-risk set. Function $L_{n}^{\left(2\right)}(\psi^{\left(2\right)},\Theta^{\left(2\right)})$ is similarly defined for the imaging-based interaction model.

The sparse penalty for the G–E model is defined as:

$$R_{a}^{\left(1\right)}(\psi^{\left(1\right)},\lambda_{1})=\sum_{j=1}^{r}\left[\rho_{a}\left(∥b_{j}^{\left(1\right)}{∥}_{2},\sqrt{q+1}\lambda_{1}\right)+\rho_{a}\left(b_{j,-1}^{\left(1\right)},\lambda_{1}\right)\right],$$

where $b_{j,-1}^{\left(1\right)}$ is a sub-vector of $b_{j}^{\left(1\right)}$ with the first element removed, and $∥\cdot {∥}_{2}$ denotes the $L_{2}$-norm for vector. Function $\rho_{a}(x;\lambda )=\int_{0}^{\left|x\right|}{\left(\lambda -t/a\right)}_{+}dt$ denotes the minimax concave penalty (MCP). For any *p*-dimensional vector x, let $\rho_{a}(x;\lambda )=\sum_{j=1}^{p}\rho_{a}(x_{j};\lambda )$. Overall, $R_{a}(\psi^{\left(1\right)},\lambda_{1})$ represents the sparse-group penalty (Simon *et al.* 2013). Here, all the effects associated with a G variable, including its main effect and interactions, are treated as a group. If a group is not selected, the corresponding G variable and interactions are excluded. Conversely, the G variable is considered as important, and the importance of related interactions is further examined. This penalty ensures the “main effects, interactions” variable selection heredity. Penalty $R_{a}^{\left(2\right)}(\psi^{\left(2\right)},\lambda_{2})$ in imaging-based model shares a similar spirit.

The final term establishes a connection between the G–E interaction model and the imaging data-based interaction model. By capturing the relationships among various G variables and imaging features, this term enhances the similarity of their predictive contributions and enables implicit information sharing between the two models. $c_{j,j^{\prime }}$ quantifies the overlap in information between the *j*th G variable and the $j^{\prime }$th imaging variable. Here, Spearman’s rank correlation is used with $c_{j,j^{\prime }}=|c_{j,j^{\prime }}^{\text{corr}}|I(|c_{j,j^{\prime }}^{\text{corr}}|>c^{\text{corr}})$, where $c_{j,j^{\prime }}^{\text{corr}}$ denotes the Spearman correlation between $z_{\cdot ,j}$ and $x_{\cdot ,j^{\prime }}$, and $c^{\text{corr}}$ denotes the cutoff used to eliminate a large proportion of spurious correlations. In our numerical studies, we set it to the 0.9 quantile of $\left|c_{j,j^{\prime }}\right|$’s. The use of different cutoffs is also investigated in simulations, demonstrating that the results remain robust within a certain range of values. We refer to Tables 15 and 16, available as supplementary data at *Bioinformatics* online, for more details. $\sum_{i=0}^{n}[B_{j}(z_{i,j},d_{i})-U_{j^{\prime }}{\left(x_{i,j^{\prime }},d_{i})\right]}^{2}\;$ measures the similarity between the effects (comprising main effects and associated interaction effects) of the *j*th G variable and the $j^{\prime }$th imaging variable. This penalty aligns with the concept of the “agreement penalty” in collaborative regression (Gross and Tibshirani 2015). However, it fosters “coordinate-wise” similarity, offering a more nuanced and appropriate impact for the G–E analysis.

**Figure 2.**
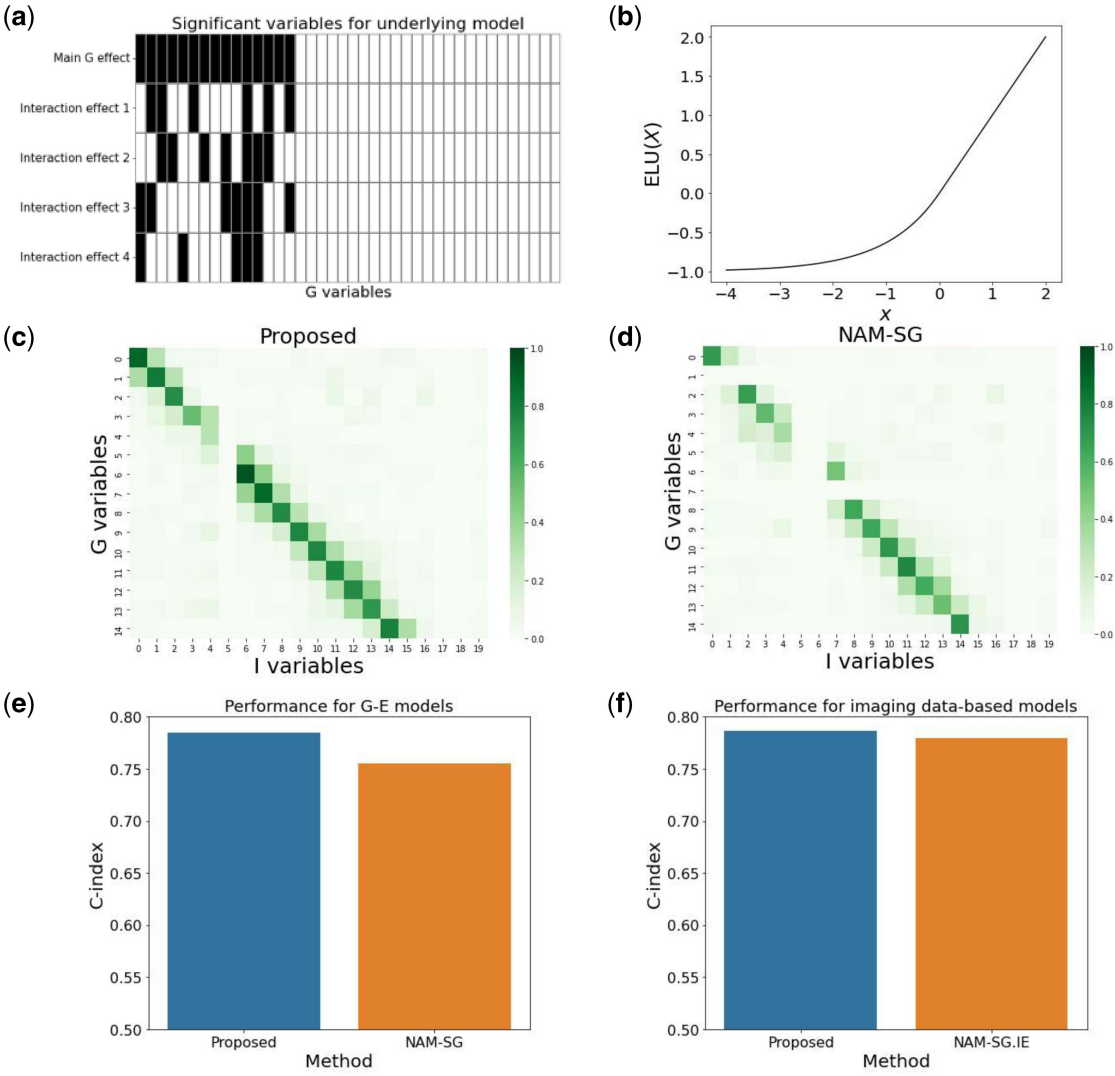
Toy example. (a) Map for important G variables and interaction terms. (b) ELU(x) function used in additive terms. (c) Heatmap of Spearman correlations between the effects of G variables and imaging variables for the proposed method. (d) Heatmap based on NAM-SG. (e) Performance comparison for G–E models. (f) Performance comparison for imaging data-based models.

**A toy example:** To gain insight into the proposed penalized estimation, we analyze a simple simulated example that resembles Example 1 in Section 3 but has reduced dimensionality r=40, p=20, and a smaller sample size n=100. The relationship between $z_{i}$ and $x_{i}$ is assumed to be $x_{i}=H^{*}z_{i}+\epsilon_{i}$, where $H^{*}$ has a banded structure. We analyze the data using the proposed method and compare it with the NAM-SG and NAM-SG.IE methods, which employ similar NAM architectures but separately estimate the G–E and imaging data-based interaction models. As shown in Fig. 2, the proposed method enhances the similarity between the effects of genetic and imaging variables due to the coordinate-wise agreement penalty, and achieves improved predictive performance as measured by the *C*-index. These results partially highlight the mechanism and advantages of the proposed assisted-learning method, which leverages information from imaging features.

### 2.3 Computation

We adopt the proximal gradient descent algorithm, a variant of traditional gradient descent that effectively accommodates nondifferentiable penalty terms. This approach has also been employed in the penalized DNN literature (Lemhadri *et al.* 2021). Specifically, we reformulate the original objective function by separating the differentiable component from the nondifferentiable penalty terms. The model parameters are then iteratively updated using gradient descent and proximal shrinkage. Detailed update steps are provided in Section 1.1, available as supplementary data at *Bioinformatics* online. The computation time of the proposed method is moderate in our numerical studies, as presented in Table 12, available as supplementary data at *Bioinformatics* online. There are four tuning parameters, $\lambda_{1}$, $\lambda_{2}$, *a*, and $\lambda_{3}$, in objective function (4). Following the literature, we set the scale parameter for MCP to a=3. The remaining tuning parameters are selected using a criterion similar to the Bayesian information criterion (BIC). Its formulation is presented in Section 1.2, available as supplementary data at *Bioinformatics* online.

## 3 Simulation

Data are generated as follows. (a) We consider (r,p,q)=(300,200,4), and n∈{500,1000}. E variables $d_{i}∼N(0_{q},\Sigma_{q}(0.3))$, where $\Sigma_{h}(\rho )$ is a h×h matrix with an autoregressive structure in that ${\left[\Sigma_{h}\right]}_{i,j}=\rho^{\left|i-j\right|}$. Similarly, G variables $z_{i}∼N(0_{r},\Sigma_{r}(0.5))$. (b) The following four settings are considered for the relationship between G and imaging variables. (b.1) Banded structure. We adopt the linear model that $x_{i}=H^{*}z_{i}+\epsilon_{i}$ with $H^{*}$ being the (p×r)-dimensional coefficient matrix, and $\epsilon_{i}$ being the error term. $H^{*}$ has a banded structure matrix, under which all the diagonal elements equal one, ${\left[H^{*}\right]}_{j,j^{\prime }}=0.3$ for $|j-j^{\prime }|=1$, and the other elements are zero. The error terms are generated as $\epsilon_{i}∼0.05N(0_{p},\Sigma_{p}(0.3))$. (b.2) Diagonal structure. The overall set-ups are similar to (b.1) except that $H^{*}$ is the diagonal structure with all its diagonal elements equal one. (b.3) Randomly-set structure. The overall set-ups are similar to (b.1) except that $H^{*}$ has the randomly-set structure, under which all its diagonal elements equal one, the randomly selected 5% off-diagonal elements are non-zero with their values sampled from Uniform(0.3, 0.6), and the other elements are zero. (b.4) Nonlinear structure. We consider the nonlinear relationship $x_{i}=\tanh(z_{i}H^{*})+\epsilon_{i}$ with tanh(x)={ exp(x)−exp(−x)}/{ exp(x)+exp(−x)}. The setups for $H^{*}$ and $\epsilon_{i}$ are parallel to those in (b.1). (c) The baseline hazard function for the G–E model is $h_{0}^{\left(1\right)}(t)=\text{exp}\;\{-2\}$. Correspondingly, the survival times are generated from the model with hazard $h_{i}^{\left(1\right)}(t)=\text{exp}\;\{-2+m_{i}(t)\}$. The censoring times are independently generated from an exponential distribution with its parameter adjusted to achieve ∼40% censoring. (d) There are 15 main G effects and 25 interaction effects. The positions of the important variables satisfy the “main effects, interactions” hierarchical heredity. For simplicity, we denote $\Phi^{\text{main}}=\{j:\beta_{j}\neq 0\}$ and $\Phi^{\text{inter}}=\{(k,j):\gamma_{k,j}\neq 0\}$ as the indexes of the important main G effects and G–E interaction effects, respectively. Besides, to give partial support for sensitive analysis, we also evaluate the scenario involving partial violation of heredity.

We consider four examples, representing different settings for hazard function. Here, Examples 1–3 consider the nonlinear cases, while Example 4 considers a linear case.

- **Example 1.** The hazard rate function for the G–E model is
  $$\begin{array}{l} f_{i}^{\left(1\right)}(t)=0.8\sum_{j\in \Phi^{\text{main}}}\text{ELU}(z_{i,j})+0.8\sum_{(j,k)\in \Phi^{\text{inter}}}\text{ELU}(d_{i,k}z_{i,j})\\ +0.8\sum_{k=1}^{q}\text{ELU}(d_{i,k}), \end{array}$$
  where $\text{ELU}(x)=xI(x\geq 0)+(e^{x}-1)I(x<0)$.
- **Example 2.** The hazard rate function for the G–E model is
  $$\begin{array}{l} f_{i}^{\left(1\right)}(t)=1.5\sum_{j\in \Phi^{\text{main}}}\;\text{sin}\;(z_{i,j})+1.5\sum_{(j,k)\in \Phi^{\text{inter}}}\;\text{sin}\;(d_{i,k}z_{i,j})\\ +1.5\sum_{k=1}^{q}\;\text{sin}\;(d_{i,k}). \end{array}$$
- **Example 3.** The hazard rate function for the G–E model is
  $$\begin{array}{l} f_{i}^{\left(1\right)}(t)=0.6\sum_{j\in \Phi^{\text{main}}}\tanh(z_{i,j})+\sum_{(j,k)\in \Phi^{\text{inter}}}\tanh(d_{i,k}z_{i,j})\\ +0.4\sum_{k=1}^{q}\tanh(d_{i,k}). \end{array}$$
- **Example 4.** The hazard rate function for the G–E model is
  $$f_{i}^{\left(1\right)}(t)=\sum_{j\in \Phi^{\text{main}}}\beta_{j}z_{i,j}+\sum_{(j,k)\in \Phi^{\text{inter}}}\gamma_{k,j}w_{i,k}z_{i,j}+\sum_{k=1}^{q}\alpha_{k}w_{i,k},$$

where $\beta_{j}$’s, $\gamma_{k,j}$’s, and $\alpha_{k}$’s are randomly generated from Uniform(0.5,1).

Beyond the proposed method, we also explore alternative methods for comparison: (a) NAM-SG, which adopts the NAM architecture combined with sparse-group penalized estimation. This method is conceptually similar to the proposed approach but does not incorporate imaging features. (b) NAM-MCP, which is analogous to NAM-SG but uses the MCP penalty for variable selection. Consequently, it does not guarantee the “main effects, interactions” heredity. (c) NAM-CR, which employs a model structure similar to the proposed method but adopts a collaborative regression approach (Gross and Tibshirani 2015) for joint estimation. As a multi-modal method, NAM-CR serves as the most comparable alternative. (d) AM-Bspline, which is a high-dimensional additive model based on B-spline expansions (Gaïffas and Guilloux 2012). The group MCP penalty is adopted to facilitate variable selection. (e) Linear-SG, which applies sparse-group penalized linear Cox regression, serves as a general benchmark. (f) Linear-IA, which is the pathological imaging-assisted G–E analysis method (Fang *et al.* 2023). (g) DeepGE, which refers to the method in Wu *et al.* (2023). (h) Dense, which employs the same G–E model as the proposed method but omits penalization, and therefore, does not support variable selection. (i) MA, which is a marginal approach. We analyze one G factor, all E factors, and their interactions at a time, and *P*-values are used to identify significant main effects and interactions. Unlike the other methods, MA is structurally different but is included due to its widespread use as a comparative benchmark.

In addition, although the focus of the proposed method lies in G–E interaction analysis, an imaging-based interaction model is also estimated as a “byproduct”. To gain further insights, we also evaluate the performance of this model. For comparison, we consider NAM-SG.IE, NAM-MCP.IE, NAM-CR, Linear-SG.IE, Linear-IA, DeepGE.IE, and Dense.IE as alternatives. Among these, NAM-SG.IE, NAM-MCP.IE, Linear-SG.IE, DeepGE.IE, and Dense.IE are developed similarly to the alternatives for the G–E model, with G variables replaced by imaging variables. NAM-CR and Linear-IA, similar to the proposed approach, simultaneously estimate both models, enabling direct comparison. As the imaging-based interaction model is implicitly defined, our evaluation primarily focuses on prediction.

Summary results based on 100 replicates are provided in Table 1 and Section 3, available as supplementary data at *Bioinformatics* online. For G–E interaction analysis, the proposed method demonstrates competitive performance in both variable selection and prediction across the whole spectrum of simulations. In terms of variable selection, it identifies more important G variables and G–E interactions while maintaining a low number of false positives. It also has competitive prediction performance. We observed that the proposed method generally outperformed NAM-SG, NAM-MCP, AM-Bspline, Linear-SG, Linear-IA, DeepGE, and Dense by a moderate margin. This superiority is likely due to its NAM architecture and sparse-group penalty. Furthermore, our approach surpassed both NAM-SG and NAM-CR, underscoring its effectiveness in leveraging information from imaging features. For the imaging-based interaction model, the proposed method also demonstrated competitive prediction performance. Additionally, in the linear case (Example 4), the proposed method’s performance was only slightly worse than the linear alternatives (Linear-SG and Linear-IA), indicating its capacity to handle such relatively simple scenarios.

**Table 1.** Simulation results when the relationship between G and imaging variables has a banded structure (b.1).^a^

	Method	Main effects (M)		Interactions (I)		*C*-index
		TP	FP	TP	FP	
	Proposed	14.2 (1.2)	0.6 (2.3)	23.9 (1.5)	3.5 (4.1)	0.897 (0.018)
	NAM-SG	13.6 (1.7)	1.6 (2.1)	21.0 (3.3)	4.7 (3.6)	0.868 (0.026)
	NAM-MCP	10.3 (3.0)	2.5 (3.0)	13.0 (3.9)	12.9 (10.7)	0.795 (0.037)
	NAM-CR	14.0 (1.2)	0.3 (0.9)	23.0 (1.9)	3.3 (2.2)	0.883 (0.018)
	AM-Bspline	11.8 (2.4)	15.6 (5.8)	12.7 (3.0)	27.5 (8.8)	0.734 (0.025)
	Linear-SG	12.2 (1.5)	1.1 (2.0)	17.5 (2.5)	3.6 (3.9)	0.775 (0.021)
	Linear-IA	12.4 (1.6)	0.3 (0.6)	18.4 (3.1)	1.7 (1.6)	0.783 (0.019)
	DeepGE	5.8 (2.9)	9.0 (8.0)	7.5 (3.8)	15.5 (13.8)	0.679 (0.049)
	Dense	–	–	–	–	0.630 (0.007)
Example 1	MA	13.7 (1.0)	3.6 (2.0)	13.6 (2.5)	23.5 (5.9)	–
	Proposed	11.3 (2.0)	1.2 (2.4)	14.2 (3.9)	4.2 (4.3)	0.793 (0.026)
	NAM-SG	8.7 (3.4)	1.9 (3.0)	9.1 (4.7)	3.6 (5.0)	0.748 (0.032)
	NAM-MCP	7.6 (2.5)	1.5 (1.9)	5.3 (2.7)	9.0 (7.2)	0.719 (0.028)
	NAM-CR	10.7 (2.2)	2.2 (2.6)	11.3 (3.4)	4.4 (4.0)	0.768 (0.023)
	AM-Bspline	13.5 (1.1)	23.9 (7.9)	9.3 (2.9)	28.1 (8.5)	0.712 (0.017)
	Linear-SG	8.8 (1.9)	2.0 (3.8)	7.6 (3.1)	4.0 (6.0)	0.740 (0.024)
	Linear-IA	8.1 (1.6)	0.6 (1.4)	7.0 (2.6)	1.7 (2.4)	0.743 (0.021)
	DeepGE	5.3 (3.0)	9.5 (5.3)	5.2 (3.2)	15.7 (9.3)	0.671 (0.048)
	Dense	–	–	–	–	0.595 (0.006)
Example 2	MA	14.5 (0.6)	3.7 (2.0)	6.8 (2.4)	19.7 (5.1)	–
	Proposed	12.2 (1.8)	0.7 (1.1)	21.0 (3.5)	3.6 (2.4)	0.815 (0.033)
	NAM-SG	10.1 (2.8)	2.0 (3.1)	15.6 (5.0)	4.5 (4.5)	0.764 (0.041)
	NAM-MCP	4.2 (2.8)	2.5 (3.5)	10.2 (4.1)	13.3 (12.2)	0.689 (0.039)
	NAM-CR	11.6 (1.7)	2.5 (4.0)	19.2 (3.4)	6.0 (6.0)	0.790 (0.029)
	AM-Bspline	8.5 (2.5)	22.8 (8.4)	16.2 (4.0)	28.6 (8.2)	0.680 (0.032)
	Linear-SG	9.2 (2.1)	2.4 (4.4)	14.3 (3.5)	5.3 (6.5)	0.749 (0.036)
	Linear-IA	9.6 (2.0)	0.4 (1.0)	15.4 (3.8)	2.5 (2.3)	0.765 (0.031)
	DeepGE	5.1 (2.4)	11.0 (8.7)	6.9 (3.6)	18.0 (14.0)	0.648 (0.058)
	Dense	–	–	–	–	0.604 (0.006)
Example 3	MA	9.9 (1.8)	2.9 (1.6)	16.2 (2.0)	16.8 (3.9)	–
	Proposed	14.3 (0.8)	0.1 (0.5)	23.9 (1.5)	2.8 (2.0)	0.906 (0.015)
	NAM-SG	13.7 (1.7)	1.9 (2.3)	20.5 (4.4)	5.1 (4.4)	0.875 (0.033)
	NAM-MCP	10.1 (2.7)	2.0 (2.4)	11.2 (3.6)	13.1 (11.4)	0.786 (0.037)
	NAM-CR	13.3 (1.4)	1.5 (4.2)	21.6 (2.9)	4.8 (6.8)	0.876 (0.030)
	AM-Bspline	11.3 (2.4)	22.0 (8.1)	11.1 (3.0)	30.0 (9.7)	0.697 (0.019)
	Linear-SG	14.2 (1.0)	1.2 (4.5)	23.2 (2.5)	3.9 (6.3)	0.905 (0.029)
	Linear-IA	14.7 (0.7)	0.1 (0.5)	24.3 (1.6)	1.5 (1.6)	0.921 (0.018)
	DeepGE	6.8 (3.0)	7.2 (7.3)	8.4 (4.2)	12.7 (12.6)	0.729 (0.065)
	Dense	–	–	–	–	0.614 (0.006)
Example 4	MA	13.5 (1.2)	3.8 (1.8)	14.9 (2.2)	22.9 (6.2)	–

a n=500 . In each cell, mean (SD) based on 100 replicates.

## 4 Data analysis

We construct G–E interaction models using lung and skin cancer data from The Cancer Genome Atlas Program (TCGA).

### 4.1 Lung cancer data analysis

Lung cancer is the leading cause of cancer-related mortality and the second most frequently diagnosed cancer in the United States. Lung adenocarcinoma (LUAD) and lung squamous cell carcinoma (LUSC) are prevalent subtypes of non-small cell lung cancer (NSCLC) that are often classified and treated similarly. This study analyzes LUAD and LUSC data. The outcome variable is the overall survival, which is right-censored. For G variables, we consider mRNA gene expressions, measured using the Illumina HiSeq RNAseq V2 platform. For E variables, we include clinical and demographic factors: age, sex, smoking history (Smoking), and the American Joint Committee on Cancer pathologic tumor stage (Stage), following previous research. For imaging variables, we use numerical features derived from pathological images. Diagnostic slides are obtained from the TCGA portal and segmented into smaller sub-images. Representative sub-images are filtered based on the presence of relevant regions. CellProfiler was then used to extract numerical features—including holistic, textural, and geometric descriptions for images—from the selected sub-images. This pipeline has been applied in several published studies (Xu *et al.* 2019).

After matching the three parts of the data and performing necessary data cleaning and processing, the dataset available for downstream analysis comprises measurements on 18 284 G variables, four E variables, and 226 imaging variables from 895 subjects. Among these, 355 subjects died during follow-up, with survival times ranging from 0 to 173 months, with a median of 19 months. For the remaining 540 censored subjects, censored times range from 0 to 238 months, with a median of 22 months. Due to the high dimensionality of the G variables and limited sample size, a supervised marginal screening procedure is applied to select the top 300 G variables. The analysis reveals small to moderate correlations between the G and imaging variables, as shown in Fig. 1, available as supplementary data at *Bioinformatics* online.

We analyze the data using the proposed method. For the G–E interaction model, a total of 12 main effects and 33 interactions are identified, as presented in Table 2. A literature review suggests that the identification results are sensible. For example, gene BTBD3 has been reported to be associated with the response to platinum-based chemotherapy in lung cancer patients. Gene FLRT3 is among the differentially expressed genes related to the mRNA stemness index in tumors and is linked to the overall survival of LUSC patients. Expression levels of the gene INTS7 are significantly elevated in LUAD tissues, and higher *INTS7* expression is associated with poorer patient prognosis. The gene KBTBD2 promotes cancer proliferation and migration by activating the epidermal growth factor receptor signaling pathway. Gene TRIB3 is highly expressed in lung cancer tissues and is associated with tumor grade and metastasis, promoting lung cancer progression by activating β-catenin signaling. We also present the identification results for the imaging-based interaction model in Table 13, available as supplementary data at *Bioinformatics* online. A total of 18 main effects and 40 interaction effects are identified, providing some biological insights.

**Table 2.** Lung cancer data analysis: identification results for G–E interaction model.^a^

Main G	Interactions			
	Age	Sex	Smoking	Stage
BTBD3	*	*	*	*
FRA10AC1	*			*
FLRT3	*		*	
INTS7		*	*	*
INTS9	*	*	*	*
KBTBD2		*		*
MESDC1	*	*		*
RAB35	*	*		*
STK24		*	*	*
TCTA	*		*	*
TRIB3	*		*	
P1P373C6	*	*		

a Asterisks indicate selected variables.

The data are also analyzed using alternative methods. The detailed identification results are omitted, while we present the overlapping identifications and corresponding RV (Smilde *et al.* 2009) coefficients between the proposed method and these alternatives in Table 14, available as supplementary data at *Bioinformatics* online. RV coefficient quantifies the overlapping information between two data matrices, as detailed in Section 3.1, available as supplementary data at *Bioinformatics* online. We observe that, based on the number of overlapping identifications and RV coefficients, the proposed method exhibits small to moderate overlap with the alternative methods. We also evaluate the prediction performance of these different methods. Specifically, we randomly split the data into training and testing datasets, with a sample size ratio of 4:1. The model is estimated using the training dataset, and prediction performance is evaluated on the testing dataset. Among 100 random splits, the proposed method has an average *C*-index of 0.614, compared to 0.583 (NAM-SG), 0.605 (NAM-MCP), 0.607 (NAM-CR), 0.582 (AM-Bspline), 0.580 (Linear-SG), 0.580 (Linear-IA), 0.584 (DeepGE), and 0.466 (Dense), demonstrating its competitive performance.

### 4.2 Skin cancer data analysis

Skin cutaneous melanoma (SKCM) is the fourth leading cause of cancer-related deaths worldwide, with advanced stages generally resulting in poor prognosis. As in the above subsection, the outcome variable is overall survival, mRNA gene expressions are used as G variables, and the extracted imaging features are used as imaging variables. For E variables, we consider age, gender, Clark level at diagnosis (Clark), Breslow depth (Breslow), and AJCC nodes pathologic stage (Stage), which are reported as sensible in the literature. After the data processing, the dataset contains the measurements on 19 037 G variables, five E variables, and 226 imaging variables for 265 subjects. Among these subjects, 126 died during follow-up. Their survival times vary from 2.6 to 340.0 months, with a median of 42.4 months. For the remaining 139 censored subjects, the observed survival times range from 0 to 301.8 months, with a median of 48.7 months. A screening procedure similar to Section 4.1 is adopted to select the top 300 G variables. Moderate correlations are identified among the G variables and imaging variables, as shown in Fig. 2, available as supplementary data at *Bioinformatics* online.

The data are analyzed using the proposed method. Table 3 represents the identification results for the G–E interaction model. A total of 10 G main effects, five E main effects, and 19 interactions are identified as important. A literature review indicates that the identification results are reasonable, with the detailed descriptions presented in Section 3.2, available as supplementary data at *Bioinformatics* online.

**Table 3.** Skin cancer data analysis: identification results for G–E interaction model.^a^

Main G	Interactions				
	Age	Sex	Breslow	Clark	Stage
ALK			*		
C2ORF42			*		
CAV3	*	*		*	*
FCGR2A	*				
GALNT9	*				
NLRC5		*	*	*	
OTOP3			*		
PKP1	*		*	*	*
SCG2	*		*		
SNORA37		*	*	*	

a Asterisks indicate selected variables.

The data are also analyzed using alternative methods. Compared to the proposed method, these approaches identified a certain number of overlapping variables, resulting in a moderate degree of information overlap. Detailed comparison results are summarized in Table 16, available as supplementary data at *Bioinformatics* online. Prediction performance is also evaluated using 100 random splits, yielding average *C*-index values of 0.682 (proposed), 0.643 (NAM-SG), 0.654 (NAM-MCP), 0.646 (NAM-CR), 0.611 (AM-Bspline), 0.557 (Linear-SG), 0.604 (Linear-IA), 0.658 (DeepGE), and 0.398 (Dense). These findings further confirm the superior predictive performance of the proposed method.

## 5 Discussion

G–E interaction model plays a crucial role in biomedical research. Beyond traditional regression-based G–E models, recent studies have explored DNN-based approaches, which offer greater flexibility for modeling complex data scenarios. However, DNN-based G–E models often face challenges due to information deficiency, underscoring the need for assisted-learning methods. This study investigates a pathological imaging-assisted neural additive model for G–E analysis. We employ an interpretable additive neural network architecture. Additionally, an assisted-learning method is proposed to facilitate information borrowing from widely accessible and highly informative pathological imaging data. Numerical studies demonstrate that the proposed method improves both identification and prediction performance compared to alternative methods.

Potential extensions include broader applications in interaction analysis, such as gene–gene interaction analysis (Cui *et al.* 2022), higher-order interaction analysis (Balasubramanian 2021), and functional interaction modeling (Liang *et al.* 2023). The assisted-learning strategy for G–E modeling can also be extended to incorporate more diverse types of auxiliary data. Additionally, it is of interest to adapt the proposed NAM architecture for interaction modeling to Bayesian neural networks (Blundell *et al.* 2015), which offer distinct advantages in quantifying uncertainty.

## Supplementary Material

btaf481_Supplementary_Data

## Data Availability

The data that support the findings in this article are openly available in TCGA (The Cancer Genome Atlas) at https://portal.gdc.cancer.gov/.
