# Supplementary material for: GE-IA-NAM: gene–environment interaction analysis via imaging-assisted neural additive model
Source: Bioinformatics. 2025 Aug 29;41(9):btaf481. doi: 10.1093/bioinformatics/btaf481 (PMC12452269; doi:10.1093/bioinformatics/btaf481)
Supplement: btaf481_Supplementary_Data [file btaf481_supplementary_data.pdf]

Supplementary Material for "Gene-environment Interaction Analysis via Pathological Imaging-assisted Neural Additive Model"

S1 Additional details for computation

S1.1 Computation details

We can reformulate the objective function $\mathcal{Q}\big(\boldsymbol{\psi}^{(1)}, \boldsymbol{\Theta}^{(1)}, \boldsymbol{\psi}^{(2)}, \boldsymbol{\Theta}^{(2)}\big)$ as

$$\begin{aligned}\mathcal{Q}\big(\boldsymbol{\psi}^{(1)}, \boldsymbol{\Theta}^{(1)}, \boldsymbol{\psi}^{(2)}, \boldsymbol{\Theta}^{(2)}\big) =& \mathcal{F}\big(\boldsymbol{\psi}^{(1)}, \boldsymbol{\Theta}^{(1)}, \boldsymbol{\psi}^{(2)}, \boldsymbol{\Theta}^{(2)}\big) \\ &+ \mathcal{R}_a^{(1)}(\boldsymbol{\psi}^{(1)}, \lambda_1) + \mathcal{R}_a^{(2)}(\boldsymbol{\psi}^{(2)}, \lambda_2),\end{aligned}$$

where the function $\mathcal{F}\big(\boldsymbol{\psi}^{(1)}, \boldsymbol{\Theta}^{(1)}, \boldsymbol{\psi}^{(2)}, \boldsymbol{\Theta}^{(2)}\big) = -\mathcal{L}_n^{(1)}(\boldsymbol{\psi}^{(1)}, \boldsymbol{\Theta}^{(1)}) - \mathcal{L}_n^{(2)}(\boldsymbol{\psi}^{(2)}, \boldsymbol{\Theta}^{(2)}) + \lambda_3 \sum_{j=1}^{r} \sum_{j'=1}^{p} c_{j,j'} \big\|\mathcal{B}_j(z_{i,j}, \mathbf{d}_i) - \mathcal{U}_{j'}(x_{i,j'}, \mathbf{d}_i)\big\|_2^2$ denotes the differentiable part for the objective function. Given the parameters in the (m)-th iteration $\{\boldsymbol{\psi}^{(1)[m]}, \boldsymbol{\Theta}^{(1)[m]}, \boldsymbol{\psi}^{(2)[m]}, \boldsymbol{\Theta}^{(2)[m]}\}$, the update in the $(m+1)$-th iteration follows that

$$\begin{aligned}
\boldsymbol{\Theta}^{(1)[m+1]} &= \boldsymbol{\Theta}^{(1)[m]} - \omega \frac{\partial \mathcal{F}\big(\boldsymbol{\psi}^{(1)[m]}, \boldsymbol{\Theta}^{(1)[m]}, \boldsymbol{\psi}^{(2)[m]}, \boldsymbol{\Theta}^{(2)[m]}\big)}{\partial \boldsymbol{\Theta}^{(1)[m]}}, \\
\tilde{\boldsymbol{\psi}}^{(1)[m+1]} &= \boldsymbol{\psi}^{(1)[m]} - \omega \frac{\partial \mathcal{F}\big(\boldsymbol{\psi}^{(1)[m]}, \boldsymbol{\Theta}^{(1)[m]}, \boldsymbol{\psi}^{(2)[m]}, \boldsymbol{\Theta}^{(2)[m]}\big)}{\partial \boldsymbol{\psi}^{(1)[m]}}, \\
\boldsymbol{\psi}^{(1)[m+1]} &= \mathrm{Prox}_a^{(1)}\left(\tilde{\boldsymbol{\psi}}^{(1)[m+1]}, \lambda_1\right), \\
\boldsymbol{\Theta}^{(2)[m+1]} &= \boldsymbol{\Theta}^{(2)[m]} - \omega \frac{\partial \mathcal{F}\big(\boldsymbol{\psi}^{(1)[m]}, \boldsymbol{\Theta}^{(1)[m]}, \boldsymbol{\psi}^{(2)[m]}, \boldsymbol{\Theta}^{(2)[m]}\big)}{\partial \boldsymbol{\Theta}^{(2)[m]}}, \\
\tilde{\boldsymbol{\psi}}^{(2)[m+1]} &= \boldsymbol{\psi}^{(2)[m]} - \omega \frac{\partial \mathcal{F}\big(\boldsymbol{\psi}^{(1)[m]}, \boldsymbol{\Theta}^{(1)[m]}, \boldsymbol{\psi}^{(2)[m]}, \boldsymbol{\Theta}^{(2)[m]}\big)}{\partial \boldsymbol{\psi}^{(2)[m]}}, \\
\boldsymbol{\psi}^{(2)[m+1]} &= \mathrm{Prox}_a^{(2)}\left(\tilde{\boldsymbol{\psi}}^{(2)[m+1]}, \lambda_2\right),
\end{aligned}$$

where ω denotes the learning rate. The first-order derivatives are computed based on the backpropagation for the neural network. For any $[r(q+1)+q]$-dimensional vector $\tilde{\boldsymbol{\psi}}^{*(1)}$ and $[p(q+1)+q]$-dimensional vector $\tilde{\boldsymbol{\psi}}^{*(2)}$, we define the functions $\mathrm{Prox}_a^{(1)}(\tilde{\boldsymbol{\psi}}^{*(1)})$ and $\mathrm{Prox}_a^{(2)}(\tilde{\boldsymbol{\psi}}^{*(2)})$ as proximal functions corresponding to penalties $\mathcal{R}_a^{(1)}$ and $\mathcal{R}_a^{(2)}$, specifically:

$$\mathrm{Prox}_a^{(1)}\left(\tilde{\boldsymbol{\psi}}^{*(1)}, \lambda_1\right) = \arg\min_{\boldsymbol{\psi}^{(1)}} \frac{1}{2}\left\|\boldsymbol{\psi}^{(1)} - \tilde{\boldsymbol{\psi}}^{*(1)}\right\|_2^2 + \mathcal{R}_a^{(1)}\left(\boldsymbol{\psi}^{(1)}, \lambda_1\right). \tag{S1.1}$$

The proximal function $\mathrm{Prox}_a^{(2)}\left(\tilde{\boldsymbol{\psi}}^{*(2)}, \lambda_2\right)$ is similarly defined.

We discuss the optimization problem (S1.1). Denote $P_a(\mathbf{b}_j^{(1)}, \lambda_1) = \rho_a\left(\|\mathbf{b}_j^{(1)}\|_2, \sqrt{q+1}\lambda_1\right) + \rho_a\left(\mathbf{b}_{j,-1}^{(1)}, \lambda_1\right)$. Recall that $\boldsymbol{\psi}^{(1)} = (\mathbf{b}_1^{(1)\top}, \ldots, \mathbf{b}_r^{(1)\top}, \boldsymbol{\gamma}^\top)^\top$ and the penalty $\mathcal{R}_a^{(1)}(\boldsymbol{\psi}^{(1)}, \lambda_1) = \sum_{j=1}^r P_a(\mathbf{b}_j^{(1)}, \lambda_1)$. Meanwhile, we denote the vector $\tilde{\boldsymbol{\psi}}^{*(1)} = (\mathbf{b}_1^{*(1)\top}, \ldots, \mathbf{b}_r^{*(1)\top}, \boldsymbol{\gamma}^{*\top})^\top$, and the solution to (S1.1) as $\widehat{\boldsymbol{\psi}}^{(1)} = (\widehat{\mathbf{b}}_1^{(1)\top}, \ldots, \widehat{\mathbf{b}}_r^{(1)\top}, \widehat{\boldsymbol{\gamma}}^\top)^\top$ mimicking $\boldsymbol{\psi}^{(1)}$. It is then observed that the optimization problem (S1.1) is separable with

$$\widehat{\boldsymbol{\gamma}} = \boldsymbol{\gamma}^*, \tag{S1.2}$$

$$\widehat{\mathbf{b}}_j^{(1)} = \arg\min_{\mathbf{b}_j^{(1)}} \frac{1}{2}\left\|\mathbf{b}_j^{(1)} - \mathbf{b}_j^{*(1)}\right\|_2^2 + P_a(\mathbf{b}_j^{(1)}, \lambda_1), \tag{S1.3}$$

Finally, following Fang et al. (2023), an iterative method is designed to solve the optimization problem (S1.3). We summarize the detailed procedures in Algorithm 1.

Algorithm 1: Computation for optimization problem (S1.3).

Input: $(q+1)$-vector $\mathbf{b}_j^{*(1)}$; tuning parameter λ_1; stopping criterion ϵ;

if $\|\,\mathrm{ST}_{-1}\left(\mathbf{b}_j^{*(1)}; \lambda_1\right)\|_2 \le \sqrt{q+1}\lambda_1$, **then**

  $\widehat{\mathbf{b}}_j^{(1)} = \mathbf{0}$;

else

  Define index set $\Psi = \{k = 1, \ldots, (q+1) : k = 1 \quad \text{or} \quad |b_{j,k}^{*(1)}| > \lambda_1\}$ and its complement Ψ^c;

  Set $\widehat{\mathbf{b}}_{j,\Psi^c}^{(1)} = \mathbf{0}$;

  Initialize: $\widehat{\mathbf{b}}_\Psi^{(1)[0]} = \mathbf{b}_{j,\Psi}^{*(1)}$.

  for *m=1,2,...* **do**

$$\widehat{\mathbf{b}}_{j,\Psi}^{(1)[m]} = \frac{\mathbf{b}_{j,\Psi}^{*(1)} - \rho_a'\left(\widehat{\mathbf{b}}_\Psi^{(1)[m-1]}; \lambda_1\right)}{1 + \rho_a'\left(\left\|\widehat{\mathbf{b}}_\Psi^{(1)[m-1]}\right\|_2; \sqrt{q+1}\lambda_1\right) / \left\|\widehat{\mathbf{b}}_\Psi^{(1)[m-1]}\right\|_2}.$$

    if $\left\|\widehat{\mathbf{b}}_\Psi^{(1)[m]} - \widehat{\mathbf{b}}_\Psi^{(1)[m-1]}\right\|_2 < \epsilon$ **then**

      terminate iteration;

    end

  end

end

Output: $(q+1)$-dimensional vector $\widehat{\mathbf{b}}_\Psi^{(1)[m]}$.

Remark:

(1) $\mathrm{ST}_{-1}(\mathbf{x}; \lambda) = (x_1, [\mathrm{sign}(\mathbf{x}_{-1}) \circ (|\mathbf{x}_{-1}| - \lambda)_+]^\top)^\top$;

(2) $\rho_a'(x; \lambda) = \mathrm{sign}(x) I(|x| < a\lambda)(\lambda - |x|/a)$.

Overall, we iterate the update steps until the stopping criteria are met.

S1.2 Definition of BIC for tuning parameter selection

For the estimated parameters $\{\widehat{\boldsymbol{\psi}}_n^{(1)}, \widehat{\boldsymbol{\Theta}}_n^{(1)}\}$ in the G–E model, the BIC is calculated as follows:

$$\mathrm{BIC}\left(\widehat{\boldsymbol{\psi}}_n^{(1)}, \widehat{\boldsymbol{\Theta}}_n^{(1)}\right) = -2\mathcal{L}_n^{(1)}(\boldsymbol{\psi}^{(1)}, \boldsymbol{\Theta}^{(1)}) + \log(n)\widehat{s}_n, \tag{S1.4}$$

where $\widehat{s}_n$ denotes the number of nonzero elements in $\boldsymbol{\psi}^{(1)}$, i.e., the total number of selected main effects and interaction terms.

S2 Additional simulation results

Table S1: Summary of network training settings for the proposed method.

Description	Setup
Size for hidden layers	(5, 5)
Activation function	ReLU
Optimizer	AdamW(lr=0.02, weight_decay=0.1)

Table S2: Simulation results when the relationship between G and imaging variables has a banded structure (b.1). $n = 1000$. In each cell, mean (sd) based on 100 replicates.

	Method	Main effects (M)		Interactions (I)		C-index
		TP	FP	TP	FP	
Example 1	Proposed	15.0(0.0)	0.0(0.0)	25.0(0.0)	2.1(2.0)	0.924(0.002)
	NAM-SG	15.0(0.2)	0.3(0.9)	24.9(0.4)	2.1(3.0)	0.919(0.006)
	NAM-MCP	14.5(0.9)	0.1(0.3)	20.9(2.2)	1.5(2.5)	0.889(0.019)
	NAM-CR	15.0(0.2)	0.0(0.0)	24.9(0.4)	0.6(0.8)	0.920(0.006)
	AM-Bspline	14.8(0.5)	14.2(9.5)	19.9(2.7)	13.2(5.1)	0.826(0.015)
	Linear-SG	14.7(0.8)	0.5(1.1)	24.0(2.1)	3.8(2.7)	0.822(0.012)
	Linear-IA	14.4(0.8)	0.0(0.1)	22.4(1.9)	0.8(0.9)	0.814(0.010)
	DeepGE	3.9(3.3)	3.3(2.7)	5.3(4.9)	4.6(4.4)	0.651(0.087)
	Dense	-	-	-	-	0.650(0.006)
	MA	14.9(0.2)	3.6(1.9)	20.6(1.8)	22.1(6.0)	-
Example 2	Proposed	14.6(0.7)	0.6(1.9)	23.5(2.4)	6.3(3.4)	0.874(0.024)
	NAM-SG	13.6(1.9)	2.6(3.7)	17.8(5.3)	5.7(7.0)	0.814(0.028)
	NAM-MCP	13.1(1.8)	0.3(0.6)	10.4(3.5)	3.6(2.9)	0.802(0.021)
	NAM-CR	12.7(1.7)	0.1(0.4)	14.5(3.2)	1.0(1.3)	0.804(0.018)
	AM-Bspline	14.9(0.4)	16.0(8.0)	16.8(2.9)	13.3(7.9)	0.804(0.017)
	Linear-SG	12.8(2.9)	1.7(2.1)	15.1(5.3)	6.5(4.1)	0.789(0.029)
	Linear-IA	8.6(2.4)	0.1(0.2)	7.4(3.3)	0.5(0.6)	0.752(0.025)
	DeepGE	5.4(2.5)	6.9(7.3)	5.3(2.8)	11.3(11.1)	0.694(0.044)
	Dense	-	-	-	-	0.610(0.006)
Example 3	MA	15.0(0.0)	3.5(2.0)	12.4(2.0)	20.2(5.5)	-
	Proposed	14.5(0.7)	0.3(0.6)	24.7(1.3)	4.3(2.3)	0.870(0.016)
	NAM-SG	14.0(1.2)	1.4(2.9)	23.7(2.0)	4.3(5.3)	0.844(0.021)
	NAM-MCP	9.3(2.3)	0.2(0.5)	19.0(3.5)	3.4(3.7)	0.803(0.034)
	NAM-CR	13.2(1.3)	0.1(0.3)	22.9(2.0)	1.2(1.6)	0.838(0.019)
	AM-Bspline	12.9(1.5)	15.1(7.2)	23.0(1.6)	10.5(5.1)	0.796(0.017)
	Linear-SG	13.4(2.1)	1.6(2.3)	22.5(4.1)	5.7(4.2)	0.823(0.032)
	Linear-IA	10.9(1.7)	0.0(0.1)	19.0(3.6)	1.1(1.0)	0.797(0.028)
	DeepGE	3.9(3.0)	3.4(2.4)	6.0(5.5)	5.2(3.3)	0.640(0.091)
	Dense	-	-	-	-	0.622(0.007)
Example 4	MA	14.0(1.1)	3.5(1.8)	22.0(1.4)	18.4(5.1)	-
	Proposed	14.9(0.3)	0.1(0.6)	24.9(0.5)	2.9(2.5)	0.928(0.008)
	NAM-SG	14.8(0.8)	1.1(2.7)	24.0(2.4)	4.1(5.4)	0.918(0.027)
	NAM-MCP	13.9(1.7)	0.2(0.5)	18.3(4.0)	3.7(6.9)	0.873(0.037)
	NAM-CR	14.5(1.0)	0.1(0.3)	24.1(1.7)	1.1(1.5)	0.918(0.021)
	AM-Bspline	14.5(0.7)	16.7(10.0)	18.3(1.9)	11.9(5.5)	0.809(0.017)
	Linear-SG	14.9(0.3)	0.2(1.3)	24.7(1.3)	1.3(2.5)	0.931(0.013)
	Linear-IA	14.9(0.2)	0.0(0.0)	24.8(0.8)	0.2(0.4)	0.933(0.007)
	DeepGE	5.9(3.7)	2.2(4.4)	8.3(6.1)	3.2(6.6)	0.731(0.102)
	Dense	-	-	-	-	0.634(0.007)
	MA	14.8(0.4)	3.7(2.0)	20.5(1.6)	23.1(6.6)	-

Table S3: Simulation results when the relationship between G and I variables has a diagonal structure (b.II). $n = 500$. In each cell, mean (sd) based on 100 replicates.

	Method	Main effects (M)		Interactions (I)		C-index
		TP	FP	TP	FP	
Example 1	Proposed	14.1(1.1)	0.2(0.7)	23.9(1.5)	2.9(2.7)	0.897(0.018)
	NAM-SG	13.7(1.6)	1.7(2.6)	21.1(2.9)	4.7(4.0)	0.868(0.024)
	NAM-MCP	10.3(2.7)	2.7(3.2)	12.6(3.6)	14.9(12.2)	0.791(0.033)
	NAM-CR	14.2(0.9)	0.3(0.6)	23.5(1.6)	2.8(2.2)	0.890(0.016)
	AM-Bspline	11.5(2.4)	19.2(7.5)	13.3(3.3)	29.2(9.8)	0.733(0.024)
	Linear-SG	12.2(1.5)	1.1(2.0)	17.5(2.5)	3.6(3.9)	0.775(0.021)
	Linear-IA	12.5(1.6)	0.2(0.5)	18.5(2.6)	1.6(1.4)	0.784(0.018)
	DeepGE	5.3(3.0)	9.3(7.7)	6.4(4.0)	15.0(14.0)	0.665(0.053)
	Dense	-	-	-	-	0.630(0.007)
	MA	13.7(1.0)	3.6(2.0)	13.6(2.5)	23.5(5.9)	-
Example 2	Proposed	11.2(2.1)	0.9(1.8)	14.0(4.1)	3.5(2.9)	0.794(0.028)
	NAM-SG	9.0(2.9)	2.6(3.9)	9.3(4.3)	4.1(4.8)	0.749(0.028)
	NAM-MCP	7.1(3.0)	1.8(2.5)	5.1(2.9)	9.4(10.5)	0.713(0.033)
	NAM-CR	12.0(1.6)	4.8(5.1)	13.2(3.1)	7.5(7.0)	0.775(0.019)
	AM-Bspline	13.2(2.3)	22.2(7.9)	9.9(3.5)	27.6(8.8)	0.716(0.022)
	Linear-SG	8.8(1.9)	2.0(3.8)	7.6(3.1)	4.0(6.0)	0.740(0.024)
	Linear-IA	8.6(1.6)	0.6(1.6)	7.8(2.9)	1.8(2.4)	0.748(0.019)
	DeepGE	4.9(2.9)	8.1(5.3)	5.0(3.1)	13.5(9.0)	0.668(0.056)
	Dense	-	-	-	-	0.595(0.006)
	MA	14.5(0.6)	3.7(2.0)	6.8(2.4)	19.7(5.1)	-
Example 3	Proposed	12.2(1.8)	0.8(1.2)	21.3(3.4)	3.8(2.2)	0.816(0.031)
	NAM-SG	10.1(2.9)	2.4(3.1)	16.1(5.5)	4.6(4.0)	0.765(0.045)
	NAM-MCP	3.7(2.5)	2.3(3.7)	9.6(4.2)	10.4(12.1)	0.684(0.042)
	NAM-CR	12.4(1.5)	3.0(4.5)	20.2(3.0)	7.3(7.1)	0.797(0.026)
	AM-Bspline	8.0(2.5)	24.0(7.8)	16.2(3.0)	30.2(8.8)	0.680(0.021)
	Linear-SG	9.2(2.1)	2.4(4.4)	14.3(3.5)	5.3(6.5)	0.749(0.036)
	Linear-IA	9.5(2.1)	0.5(1.0)	15.9(4.3)	2.2(2.0)	0.768(0.036)
	DeepGE	5.3(2.6)	11.0(7.7)	7.5(3.9)	18.8(12.9)	0.649(0.055)
	Dense	-	-	-	-	0.604(0.006)
	MA	9.9(1.8)	2.9(1.6)	16.2(2.0)	16.8(3.9)	-
Example 4	Proposed	14.2(0.9)	0.1(0.3)	23.5(1.9)	2.5(1.5)	0.904(0.018)
	NAM-SG	13.8(1.6)	2.1(2.4)	20.7(4.5)	5.7(4.2)	0.875(0.035)
	NAM-MCP	10.3(2.5)	2.7(3.0)	11.6(4.1)	14.3(11.9)	0.790(0.034)
	NAM-CR	14.0(0.9)	2.1(4.6)	22.3(2.4)	5.7(6.7)	0.886(0.028)
	AM-Bspline	11.5(2.2)	21.9(7.6)	11.6(3.2)	31.1(10.5)	0.704(0.024)
	Linear-SG	14.2(1.0)	1.2(4.5)	23.2(2.5)	3.9(6.3)	0.905(0.029)
	Linear-IA	14.5(0.8)	0.0(0.3)	24.1(1.7)	1.2(1.4)	0.920(0.018)
	DeepGE	6.6(3.2)	5.9(5.0)	8.1(4.6)	10.0(7.9)	0.725(0.068)
	Dense	-	-	-	-	0.614(0.006)
	MA	13.5(1.2)	3.8(1.8)	14.9(2.2)	22.9(6.2)	-

Table S4: Simulation results when the relationship between G and I variables has a diagonal structure (b.II). $n = 1000$. In each cell, mean (sd) based on 100 replicates.

	Method	Main effects (M)		Interactions (I)		C-index
		TP	FP	TP	FP	
Example 1	Proposed	15.0(0.1)	0.0(0.0)	25.0(0.0)	2.2(1.9)	0.923(0.002)
	NAM-SG	15.0(0.0)	0.5(1.2)	24.9(0.3)	2.6(3.1)	0.919(0.006)
	NAM-MCP	14.5(0.9)	0.1(0.2)	20.9(1.9)	1.1(1.2)	0.890(0.017)
	NAM-CR	15.0(0.1)	0.0(0.0)	25.0(0.0)	0.7(0.9)	0.922(0.003)
	AM-Bspline	14.8(0.5)	14.2(10.9)	20.2(2.1)	15.4(6.4)	0.826(0.015)
	Linear-SG	14.7(0.8)	0.5(1.1)	24.0(2.1)	3.8(2.7)	0.822(0.012)
	Linear-IA	14.4(0.8)	0.0(0.2)	22.7(2.2)	0.8(0.8)	0.814(0.011)
	DeepGE	3.7(3.6)	4.2(5.6)	4.8(5.2)	7.0(10.1)	0.646(0.083)
	Dense	-	-	-	-	0.650(0.006)
	MA	14.9(0.2)	3.6(1.9)	20.6(1.8)	22.1(6.0)	-
Example 2	Proposed	14.6(0.7)	0.8(1.7)	23.4(2.5)	6.1(3.1)	0.873(0.025)
	NAM-SG	13.8(1.7)	2.4(3.8)	18.1(4.7)	5.3(6.8)	0.819(0.027)
	NAM-MCP	12.7(1.9)	0.2(0.6)	10.7(3.3)	3.7(4.8)	0.800(0.025)
	NAM-CR	13.3(1.4)	0.1(0.5)	15.7(3.5)	1.1(1.4)	0.811(0.017)
	AM-Bspline	14.9(0.2)	16.5(9.7)	17.9(3.5)	11.0(5.8)	0.809(0.015)
	Linear-SG	12.8(2.9)	1.7(2.1)	15.1(5.3)	6.5(4.1)	0.789(0.029)
	Linear-IA	8.5(2.4)	0.0(0.2)	7.3(3.0)	0.6(0.9)	0.751(0.025)
	DeepGE	4.2(2.9)	3.9(2.4)	4.0(3.4)	6.7(3.3)	0.674(0.058)
	Dense	-	-	-	-	0.610(0.006)
	MA	15.0(0.0)	3.5(2.0)	12.4(2.0)	20.2(5.5)	-
Example 3	Proposed	14.6(0.8)	0.2(0.4)	24.7(0.9)	3.8(2.5)	0.871(0.012)
	NAM-SG	14.2(1.1)	1.6(2.8)	24.0(1.8)	4.1(5.1)	0.849(0.020)
	NAM-MCP	9.9(2.0)	0.2(0.9)	20.1(2.9)	4.6(9.8)	0.810(0.033)
	NAM-CR	13.8(0.8)	0.0(0.1)	23.7(1.5)	1.0(1.1)	0.846(0.015)
	AM-Bspline	12.9(1.4)	13.9(7.2)	22.8(1.8)	12.9(5.2)	0.795(0.017)
	Linear-SG	13.4(2.1)	1.6(2.3)	22.5(4.1)	5.7(4.2)	0.823(0.032)
	Linear-IA	11.5(1.8)	0.0(0.1)	19.6(3.6)	0.8(0.9)	0.804(0.027)
	DeepGE	4.0(3.2)	3.2(2.7)	6.2(5.1)	5.6(5.0)	0.643(0.089)
	Dense	-	-	-	-	0.622(0.007)
	MA	14.0(1.1)	3.5(1.8)	22.0(1.4)	18.4(5.1)	-
Example 4	Proposed	14.9(0.4)	0.1(0.6)	24.9(0.5)	2.3(2.2)	0.928(0.008)
	NAM-SG	14.8(0.6)	1.4(3.1)	24.0(2.1)	4.4(5.7)	0.916(0.026)
	NAM-MCP	13.5(2.0)	0.2(0.5)	17.7(3.6)	3.4(3.3)	0.866(0.036)
	NAM-CR	14.7(0.7)	0.1(0.3)	24.4(1.3)	1.3(1.7)	0.921(0.017)
	AM-Bspline	14.3(0.9)	17.1(9.9)	17.7(2.3)	14.0(8.9)	0.803(0.019)
	Linear-SG	14.9(0.3)	0.2(1.3)	24.7(1.3)	1.3(2.5)	0.931(0.013)
	Linear-IA	14.9(0.3)	0.0(0.0)	24.9(0.6)	0.3(0.6)	0.933(0.007)
	DeepGE	4.7(3.7)	1.0(2.0)	6.1(5.4)	2.1(3.1)	0.693(0.111)
	Dense	-	-	-	-	0.634(0.007)
	MA	14.8(0.4)	3.7(2.0)	20.5(1.6)	23.1(6.6)	-

Table S5: Simulation results when the relationship between G and I variables has a randomly-set structure (b.III). $n = 500$. In each cell, mean (sd) based on 100 replicates.

	Method	Main effects (M)		Interactions (I)		C-index
		TP	FP	TP	FP	
Example 1	Proposed	14.4(0.7)	0.1(0.6)	24.0(1.4)	3.1(1.9)	0.901(0.013)
	NAM-SG	13.2(1.9)	2.4(2.9)	20.1(4.0)	6.0(4.6)	0.857(0.031)
	NAM-MCP	9.3(3.1)	2.1(2.6)	11.4(3.8)	10.7(10.0)	0.783(0.034)
	NAM-CR	13.5(1.6)	1.0(1.7)	21.4(2.9)	4.1(3.4)	0.868(0.026)
	AM-Bspline	12.2(1.6)	19.1(6.9)	13.1(2.7)	28.6(7.3)	0.736(0.020)
	Linear-SG	11.9(1.5)	1.1(1.6)	16.7(2.6)	3.5(3.3)	0.775(0.017)
	Linear-IA	11.9(1.7)	0.4(0.7)	17.1(3.1)	1.8(1.5)	0.779(0.018)
	DeepGE	4.9(2.8)	7.9(6.3)	6.4(4.0)	13.6(11.1)	0.663(0.055)
	Dense	-	-	-	-	0.630(0.008)
	MA	13.9(1.1)	3.5(2.0)	13.3(2.2)	24.1(6.6)	-
Example 2	Proposed	11.7(2.0)	0.7(1.1)	14.4(4.1)	3.6(2.8)	0.797(0.029)
	NAM-SG	8.8(3.4)	1.9(2.9)	8.7(5.2)	3.7(4.3)	0.745(0.036)
	NAM-MCP	7.4(2.6)	1.5(1.8)	5.1(2.9)	8.9(8.4)	0.718(0.033)
	NAM-CR	10.5(2.4)	1.9(2.1)	10.7(3.5)	3.9(3.1)	0.765(0.021)
	AM-Bspline	13.4(1.3)	22.1(8.3)	9.9(3.2)	28.3(8.5)	0.717(0.021)
	Linear-SG	8.9(1.8)	1.8(3.3)	8.2(2.9)	3.8(4.3)	0.745(0.024)
	Linear-IA	9.0(2.5)	1.1(2.6)	8.4(3.6)	2.2(3.7)	0.748(0.028)
	DeepGE	4.9(2.7)	10.3(9.4)	4.5(3.0)	17.6(16.8)	0.663(0.050)
	Dense	-	-	-	-	0.595(0.007)
	MA	14.5(0.6)	3.8(1.8)	6.5(2.2)	21.4(6.1)	-
Example 3	Proposed	12.3(1.7)	0.5(0.8)	21.5(2.8)	3.2(2.2)	0.821(0.026)
	NAM-SG	10.6(2.6)	3.1(4.3)	16.5(5.4)	5.3(5.4)	0.768(0.045)
	NAM-MCP	4.4(2.3)	2.3(4.1)	9.6(3.6)	10.1(12.4)	0.693(0.031)
	NAM-CR	11.5(2.2)	2.7(3.7)	18.7(3.6)	5.4(5.8)	0.788(0.039)
	AM-Bspline	7.8(2.6)	19.7(8.7)	15.8(3.9)	27.8(10.2)	0.680(0.034)
	Linear-SG	9.3(1.8)	2.4(4.3)	14.6(3.1)	5.5(6.9)	0.749(0.033)
	Linear-IA	9.5(2.1)	0.9(2.2)	15.3(3.7)	2.8(4.4)	0.763(0.034)
	DeepGE	4.7(2.3)	8.7(6.7)	6.4(3.5)	13.9(11.8)	0.642(0.054)
	Dense	-	-	-	-	0.604(0.008)
	MA	9.9(2.1)	3.0(1.5)	16.2(2.1)	17.1(5.5)	-
Example 4	Proposed	14.0(1.3)	0.6(1.7)	23.1(2.6)	3.8(4.0)	0.898(0.025)
	NAM-SG	12.7(2.2)	2.0(2.4)	18.3(4.8)	5.5(5.3)	0.853(0.042)
	NAM-MCP	9.0(2.7)	2.6(3.8)	10.2(3.6)	14.3(11.7)	0.770(0.037)
	NAM-CR	13.3(1.6)	1.7(3.0)	20.1(3.8)	5.2(5.3)	0.866(0.037)
	AM-Bspline	12.0(2.1)	20.9(7.6)	11.4(2.9)	28.0(8.9)	0.705(0.024)
	Linear-SG	14.1(1.0)	1.2(2.8)	23.3(2.4)	4.7(5.1)	0.903(0.030)
	Linear-IA	14.4(1.0)	0.2(0.6)	23.6(2.3)	2.0(2.0)	0.911(0.023)
	DeepGE	6.6(3.2)	5.0(6.1)	8.5(4.7)	9.0(9.6)	0.729(0.078)
	Dense	-	-	-	-	0.613(0.007)
	MA	13.6(1.1)	3.6(2.0)	14.9(1.8)	23.2(7.7)	-

Table S6: Simulation results when the relationship between G and I variables has a randomly-set structure (b.III). $n = 1000$. In each cell, mean (sd) based on 100 replicates.

	Method	Main effects (M)		Interactions (I)		C-index
		TP	FP	TP	FP	
Example 1	Proposed	15.0(0.0)	0.0(0.0)	25.0(0.1)	2.5(2.1)	0.922(0.003)
	NAM-SG	14.9(0.3)	0.4(0.9)	24.8(0.5)	2.6(3.2)	0.917(0.008)
	NAM-MCP	14.6(0.7)	0.0(0.0)	21.5(2.1)	1.3(2.7)	0.893(0.017)
	NAM-CR	14.8(0.5)	0.0(0.0)	24.7(1.1)	0.6(0.9)	0.912(0.012)
	AM-Bspline	14.8(0.6)	15.2(10.5)	20.1(2.1)	15.3(7.5)	0.822(0.014)
	Linear-SG	14.7(0.5)	0.3(0.6)	24.4(1.0)	3.0(1.7)	0.823(0.007)
	Linear-IA	14.2(1.0)	0.0(0.0)	22.4(2.1)	1.0(1.0)	0.813(0.010)
	DeepGE	4.1(3.4)	3.5(3.0)	5.6(5.4)	5.4(5.1)	0.660(0.079)
	Dense	-	-	-	-	0.651(0.006)
	MA	15.0(0.2)	3.9(1.9)	20.5(2.1)	22.0(5.2)	-
Example 2	Proposed	14.8(0.4)	0.8(1.3)	24.0(1.6)	7.3(3.0)	0.878(0.019)
	NAM-SG	13.0(3.0)	2.9(4.3)	16.8(5.4)	5.5(6.1)	0.806(0.040)
	NAM-MCP	12.5(2.5)	0.4(0.9)	10.2(3.5)	4.3(5.4)	0.795(0.031)
	NAM-CR	11.4(2.4)	0.2(0.8)	12.4(3.7)	0.8(1.4)	0.789(0.027)
	AM-Bspline	14.9(0.3)	15.8(7.1)	17.3(2.8)	10.6(4.8)	0.807(0.015)
	Linear-SG	12.5(2.9)	2.0(2.3)	15.4(5.6)	6.6(4.4)	0.787(0.030)
	Linear-IA	8.3(1.8)	0.1(0.8)	6.9(3.0)	0.7(2.1)	0.748(0.023)
	DeepGE	5.9(3.1)	7.0(6.0)	5.9(3.9)	11.8(9.1)	0.698(0.056)
	Dense	-	-	-	-	0.611(0.007)
	MA	15.0(0.1)	4.2(2.4)	12.0(2.6)	19.3(5.2)	-
Example 3	Proposed	14.6(0.9)	0.2(0.7)	24.8(0.8)	4.3(2.5)	0.872(0.012)
	NAM-SG	13.9(1.3)	1.4(2.3)	23.6(2.2)	3.9(4.7)	0.845(0.024)
	NAM-MCP	9.2(2.6)	0.3(0.7)	19.3(3.8)	3.3(3.8)	0.803(0.038)
	NAM-CR	12.7(1.3)	0.0(0.0)	22.7(2.0)	0.7(0.8)	0.834(0.021)
	AM-Bspline	12.8(2.0)	14.2(6.4)	23.0(1.8)	12.5(7.3)	0.792(0.021)
	Linear-SG	13.4(2.0)	1.4(2.6)	22.9(3.6)	5.6(3.8)	0.826(0.032)
	Linear-IA	11.4(2.1)	0.0(0.2)	19.3(4.1)	0.8(0.9)	0.800(0.032)
	DeepGE	5.0(3.2)	3.5(2.8)	8.1(5.5)	6.0(5.2)	0.671(0.080)
	Dense	-	-	-	-	0.620(0.007)
	MA	14.0(1.1)	3.9(2.0)	22.1(1.3)	18.5(5.0)	-
Example 4	Proposed	14.9(0.3)	0.0(0.0)	24.9(0.5)	2.4(1.8)	0.928(0.007)
	NAM-SG	14.8(0.9)	0.5(1.4)	24.4(1.7)	2.9(3.6)	0.922(0.021)
	NAM-MCP	14.0(1.6)	0.1(0.3)	18.9(3.1)	2.3(3.4)	0.879(0.031)
	NAM-CR	14.6(0.8)	0.0(0.1)	24.1(1.7)	0.7(1.1)	0.918(0.021)
	AM-Bspline	14.5(0.9)	16.5(9.3)	17.9(2.8)	12.5(6.1)	0.806(0.018)
	Linear-SG	15.0(0.3)	0.1(0.6)	24.9(0.5)	1.3(1.6)	0.933(0.007)
	Linear-IA	14.9(0.4)	0.0(0.0)	24.9(0.4)	0.2(0.5)	0.931(0.007)
	DeepGE	4.6(3.6)	0.4(0.6)	6.1(5.1)	1.3(1.4)	0.689(0.116)
	Dense	-	-	-	-	0.634(0.007)
	MA	14.9(0.4)	4.4(2.4)	20.6(2.1)	23.1(6.3)	-

Table S7: Simulation results when the relationship between G and I variables has a the nonlinear structure (b.IV). $n = 500$. In each cell, mean (sd) based on 100 replicates.

	Method	Main effects (M)		Interactions (I)		C-index
		TP	FP	TP	FP	
Example 1	Proposed	14.1(1.1)	0.1(0.4)	23.6(1.7)	3.0(1.8)	0.895(0.018)
	NAM-SG	13.9(1.3)	2.0(2.4)	21.6(3.1)	5.1(4.1)	0.871(0.023)
	NAM-MCP	10.4(2.7)	3.3(3.2)	13.5(3.7)	16.2(13.6)	0.794(0.032)
	NAM-CR	13.8(1.1)	0.6(0.9)	22.2(2.3)	3.6(1.9)	0.879(0.020)
	AM-Bspline	11.8(1.8)	17.8(5.9)	13.1(2.6)	27.5(8.5)	0.734(0.016)
	Linear-SG	12.2(1.5)	1.1(2.0)	17.5(2.5)	3.6(3.9)	0.775(0.021)
	Linear-IA	12.1(1.5)	0.3(0.5)	18.1(3.0)	1.5(1.2)	0.780(0.019)
	DeepGE	5.3(3.1)	8.1(7.2)	6.5(4.2)	15.3(14.4)	0.663(0.060)
	Dense	-	-	-	-	0.630(0.007)
	MA	13.7(1.0)	3.6(2.0)	13.6(2.5)	23.5(5.9)	-
Example 2	Proposed	11.8(2.1)	1.5(3.1)	14.4(3.7)	4.2(4.3)	0.797(0.027)
	NAM-SG	8.8(3.5)	2.8(4.5)	8.6(4.9)	4.7(6.2)	0.744(0.033)
	NAM-MCP	7.8(2.5)	1.6(2.3)	5.2(2.5)	10.4(10.3)	0.719(0.026)
	NAM-CR	11.2(1.9)	3.9(5.0)	11.9(3.7)	7.2(8.6)	0.767(0.028)
	AM-Bspline	13.5(1.7)	22.8(7.6)	10.0(3.1)	29.7(8.0)	0.715(0.021)
	Linear-SG	8.8(1.9)	2.0(3.8)	7.6(3.1)	4.0(6.0)	0.740(0.024)
	Linear-IA	8.2(1.5)	0.6(0.9)	7.0(2.4)	1.6(1.2)	0.743(0.017)
	DeepGE	5.6(2.9)	11.1(9.6)	5.6(3.6)	18.9(16.6)	0.670(0.043)
	Dense	-	-	-	-	0.595(0.006)
	MA	14.5(0.6)	3.7(2.0)	6.8(2.4)	19.7(5.1)	-
Example 3	Proposed	12.3(1.6)	1.1(1.4)	21.2(3.1)	3.8(2.0)	0.816(0.032)
	NAM-SG	9.7(2.8)	1.7(2.9)	15.1(5.2)	3.7(4.2)	0.760(0.044)
	NAM-MCP	3.8(2.2)	2.2(3.2)	9.7(4.0)	11.3(10.0)	0.685(0.038)
	NAM-CR	11.7(1.9)	2.9(5.0)	18.7(3.3)	6.8(8.2)	0.787(0.030)
	AM-Bspline	8.2(2.6)	21.6(8.9)	16.0(3.5)	27.4(9.5)	0.683(0.034)
	Linear-SG	9.2(2.1)	2.4(4.4)	14.3(3.5)	5.3(6.5)	0.749(0.036)
	Linear-IA	9.2(2.1)	0.4(0.6)	15.1(3.7)	1.9(1.9)	0.762(0.033)
	DeepGE	4.1(2.1)	8.7(7.4)	5.6(3.4)	14.3(12.3)	0.629(0.053)
	Dense	-	-	-	-	0.604(0.006)
	MA	9.9(1.8)	2.9(1.6)	16.2(2.0)	16.8(3.9)	-
Example 4	Proposed	14.3(0.9)	0.2(0.7)	23.5(1.8)	2.8(2.1)	0.905(0.016)
	NAM-SG	13.7(1.3)	2.0(2.8)	20.2(3.9)	5.1(4.1)	0.873(0.028)
	NAM-MCP	10.3(2.8)	2.8(3.3)	11.7(4.1)	16.8(13.9)	0.787(0.040)
	NAM-CR	13.9(1.1)	1.8(4.7)	22.2(2.5)	5.4(6.8)	0.887(0.029)
	AM-Bspline	11.0(2.8)	21.3(7.8)	10.7(3.3)	27.4(9.0)	0.696(0.030)
	Linear-SG	14.2(1.0)	1.2(4.5)	23.2(2.5)	3.9(6.3)	0.905(0.029)
	Linear-IA	14.3(0.9)	0.1(0.4)	23.8(1.8)	1.5(1.5)	0.914(0.018)
	DeepGE	7.1(2.8)	3.8(5.2)	9.8(5.1)	7.2(7.8)	0.747(0.072)
	Dense	-	-	-	-	0.614(0.006)
	MA	13.5(1.2)	3.8(1.8)	14.9(2.2)	22.9(6.2)	-

Table S8: Simulation results when the relationship between G and I variables has a the nonlinear structure (b.IV). $n = 1000$. In each cell, mean (sd) based on 100 replicates.

	Method	Main effects (M)		Interactions (I)		C-index
		TP	FP	TP	FP	
Example 1	Proposed	15.0(0.0)	0.0(0.0)	25.0(0.1)	2.9(2.2)	0.923(0.002)
	NAM-SG	15.0(0.0)	0.4(1.2)	24.8(0.8)	2.6(3.2)	0.918(0.009)
	NAM-MCP	14.5(1.1)	0.1(0.6)	20.9(2.4)	1.8(2.9)	0.889(0.021)
	NAM-CR	15.0(0.1)	0.0(0.0)	24.9(0.3)	0.6(0.8)	0.919(0.006)
	AM-Bspline	14.8(0.4)	13.5(8.2)	19.9(2.4)	14.4(5.3)	0.825(0.014)
	Linear-SG	14.7(0.8)	0.5(1.1)	24.0(2.1)	3.8(2.7)	0.822(0.012)
	Linear-IA	14.1(1.2)	0.0(0.0)	22.0(1.9)	0.6(0.8)	0.810(0.012)
	DeepGE	7.1(4.2)	5.9(6.1)	10.0(6.1)	11.4(12.9)	0.719(0.081)
	Dense	-	-	-	-	0.650(0.006)
	MA	14.9(0.2)	3.6(1.9)	20.6(1.8)	22.1(6.0)	-
Example 2	Proposed	14.6(0.9)	0.4(0.6)	23.4(2.5)	5.6(3.2)	0.874(0.023)
	NAM-SG	13.9(1.4)	2.7(3.9)	17.6(4.7)	5.7(6.6)	0.816(0.023)
	NAM-MCP	12.7(1.7)	0.4(0.6)	10.5(3.2)	4.6(4.9)	0.798(0.022)
	NAM-CR	13.0(1.3)	0.2(0.6)	15.4(3.7)	1.1(1.7)	0.809(0.017)
	AM-Bspline	14.9(0.4)	14.2(5.3)	17.1(3.3)	12.1(6.2)	0.805(0.019)
	Linear-SG	12.8(2.9)	1.7(2.1)	15.1(5.3)	6.5(4.1)	0.789(0.029)
	Linear-IA	8.5(2.2)	0.0(0.0)	7.1(3.1)	0.5(0.9)	0.750(0.027)
	DeepGE	4.1(3.0)	4.3(4.2)	4.2(3.5)	7.6(7.5)	0.674(0.057)
	Dense	-	-	-	-	0.610(0.006)
	MA	15.0(0.0)	3.5(2.0)	12.4(2.0)	20.2(5.5)	-
Example 3	Proposed	14.6(0.7)	0.3(0.5)	24.6(1.2)	4.4(3.1)	0.870(0.016)
	NAM-SG	14.1(1.1)	1.8(2.8)	23.6(2.5)	5.2(6.0)	0.845(0.026)
	NAM-MCP	9.2(2.5)	0.3(0.7)	19.2(3.4)	4.2(6.6)	0.802(0.036)
	NAM-CR	13.3(1.2)	0.0(0.0)	23.2(1.9)	1.0(1.0)	0.840(0.019)
	AM-Bspline	12.8(1.6)	14.6(8.4)	23.1(1.6)	11.5(5.5)	0.794(0.018)
	Linear-SG	13.4(2.1)	1.6(2.3)	22.5(4.1)	5.7(4.2)	0.823(0.032)
	Linear-IA	10.9(2.1)	0.0(0.0)	18.9(4.0)	1.0(1.2)	0.797(0.030)
	DeepGE	4.2(3.0)	4.1(4.1)	6.6(5.2)	7.1(6.9)	0.643(0.086)
	Dense	-	-	-	-	0.622(0.007)
	MA	14.0(1.1)	3.5(1.8)	22.0(1.4)	18.4(5.1)	-
Example 4	Proposed	14.9(0.3)	0.1(0.7)	24.9(0.5)	2.4(2.2)	0.929(0.008)
	NAM-SG	14.8(0.8)	1.3(3.9)	24.3(2.1)	3.8(6.3)	0.919(0.026)
	NAM-MCP	13.7(2.0)	0.1(0.3)	18.2(3.9)	3.2(3.7)	0.870(0.039)
	NAM-CR	14.6(0.8)	0.2(0.7)	24.2(1.7)	1.0(1.8)	0.919(0.021)
	AM-Bspline	14.4(0.8)	15.9(9.2)	17.5(2.1)	11.2(5.4)	0.806(0.018)
	Linear-SG	14.9(0.3)	0.2(1.3)	24.7(1.3)	1.3(2.5)	0.931(0.013)
	Linear-IA	14.8(0.4)	0.0(0.0)	24.7(1.1)	0.2(0.4)	0.931(0.011)
	DeepGE	4.7(3.7)	1.5(1.9)	6.2(5.3)	2.7(4.1)	0.695(0.104)
	Dense	-	-	-	-	0.634(0.007)
	MA	14.8(0.4)	3.7(2.0)	20.5(1.6)	23.1(6.6)	-

Table S9: C-index results for imaging data-based interaction model. $n = 500$. In each cell, mean (sd) based on 100 replicates.

Relationship between G and I variables	Method	Example 1	Example 2	Example 3	Example 4
(b.I) Banded structure	Proposed	0.881(0.008)	0.816(0.017)	0.815(0.017)	0.893(0.009)
	NAM-SG.IE	0.868(0.018)	0.785(0.018)	0.800(0.020)	0.889(0.014)
	NAM-MCP.IE	0.803(0.014)	0.734(0.015)	0.730(0.017)	0.814(0.014)
	NAM-CR	0.879(0.011)	0.798(0.013)	0.812(0.018)	0.896(0.010)
	Linear-SG.IE	0.793(0.011)	0.771(0.018)	0.795(0.026)	0.906(0.009)
	Linear-IA.IE	0.792(0.013)	0.757(0.022)	0.778(0.038)	0.906(0.011)
	DeepGE.IE	0.732(0.021)	0.732(0.016)	0.716(0.040)	0.825(0.026)
	Dense.IE	0.672(0.006)	0.633(0.008)	0.648(0.007)	0.667(0.009)
(b.II) Diagonal structure	Proposed	0.889(0.012)	0.796(0.021)	0.797(0.020)	0.896(0.022)
	NAM-SG.IE	0.872(0.019)	0.770(0.023)	0.785(0.027)	0.886(0.020)
	NAM-MCP.IE	0.771(0.011)	0.706(0.012)	0.703(0.013)	0.766(0.014)
	NAM-CR	0.901(0.011)	0.805(0.016)	0.829(0.019)	0.917(0.009)
	Linear-SG.IE	0.798(0.014)	0.762(0.021)	0.801(0.029)	0.926(0.009)
	Linear-IA.IE	0.796(0.015)	0.754(0.021)	0.798(0.029)	0.925(0.009)
	DeepGE.IE	0.711(0.025)	0.722(0.020)	0.700(0.030)	0.812(0.038)
	Dense.IE	0.653(0.007)	0.615(0.007)	0.625(0.007)	0.641(0.007)
(b.III) Randomly-set structure	Proposed	0.700(0.021)	0.673(0.018)	0.650(0.025)	0.706(0.021)
	NAM-SG.IE	0.669(0.026)	0.659(0.022)	0.629(0.024)	0.693(0.024)
	NAM-MCP.IE	0.680(0.011)	0.644(0.013)	0.638(0.013)	0.681(0.013)
	NAM-CR	0.682(0.031)	0.662(0.022)	0.633(0.031)	0.698(0.027)
	Linear-SG.IE	0.635(0.024)	0.654(0.022)	0.609(0.037)	0.687(0.031)
	Linear-IA.IE	0.629(0.025)	0.647(0.023)	0.574(0.050)	0.683(0.033)
	DeepGE.IE	0.631(0.022)	0.645(0.018)	0.607(0.029)	0.672(0.018)
	Dense.IE	0.655(0.008)	0.624(0.007)	0.632(0.009)	0.655(0.011)
(b.IV) Nonlinear structure	Proposed	0.848(0.011)	0.826(0.017)	0.814(0.013)	0.859(0.012)
	NAM-SG.IE	0.841(0.019)	0.800(0.031)	0.801(0.027)	0.854(0.023)
	NAM-MCP.IE	0.775(0.015)	0.747(0.020)	0.733(0.021)	0.778(0.015)
	NAM-CR	0.852(0.014)	0.819(0.019)	0.818(0.017)	0.867(0.014)
	Linear-SG.IE	0.775(0.015)	0.800(0.020)	0.803(0.033)	0.873(0.010)
	Linear-IA.IE	0.776(0.014)	0.799(0.018)	0.802(0.032)	0.876(0.008)
	DeepGE.IE	0.701(0.025)	0.764(0.012)	0.724(0.023)	0.789(0.023)
	Dense.IE	0.668(0.007)	0.642(0.007)	0.652(0.008)	0.661(0.008)

Table S10: C-index results for imaging data-based interaction model. $n = 1000$. In each cell, mean (sd) based on 100 replicates.

Relationship between G and I variables	Method	Example 1	Example 2	Example 3	Example 4
(b.I) Banded structure	Proposed	0.900(0.004)	0.852(0.019)	0.845(0.021)	0.907(0.006)
	NAM-SG.IE	0.897(0.005)	0.812(0.021)	0.827(0.029)	0.899(0.023)
	NAM-MCP.IE	0.881(0.006)	0.816(0.017)	0.814(0.020)	0.886(0.026)
	NAM-CR	0.896(0.008)	0.797(0.024)	0.823(0.025)	0.899(0.020)
	Linear-SG.IE	0.810(0.008)	0.797(0.015)	0.820(0.015)	0.914(0.006)
	Linear-IA.IE	0.808(0.009)	0.756(0.026)	0.796(0.028)	0.915(0.007)
	DeepGE.IE	0.779(0.019)	0.764(0.014)	0.759(0.020)	0.864(0.023)
	Dense.IE	0.701(0.009)	0.657(0.009)	0.679(0.007)	0.698(0.006)
(b.II) Diagonal structure	Proposed	0.921(0.002)	0.888(0.025)	0.874(0.008)	0.925(0.004)
	NAM-SG.IE	0.918(0.008)	0.825(0.017)	0.848(0.030)	0.928(0.009)
	NAM-MCP.IE	0.898(0.011)	0.815(0.022)	0.807(0.028)	0.910(0.010)
	NAM-CR	0.920(0.007)	0.838(0.021)	0.858(0.021)	0.930(0.005)
	Linear-SG.IE	0.824(0.006)	0.805(0.015)	0.836(0.023)	0.933(0.003)
	Linear-IA.IE	0.823(0.007)	0.756(0.029)	0.822(0.028)	0.933(0.003)
	DeepGE.IE	0.767(0.036)	0.759(0.019)	0.750(0.027)	0.828(0.058)
	Dense.IE	0.680(0.005)	0.634(0.006)	0.650(0.007)	0.666(0.006)
(b.III) Randomly-set structure	Proposed	0.722(0.020)	0.688(0.023)	0.655(0.034)	0.724(0.026)
	NAM-SG.IE	0.687(0.034)	0.678(0.028)	0.632(0.046)	0.720(0.027)
	NAM-MCP.IE	0.720(0.013)	0.685(0.016)	0.668(0.017)	0.732(0.017)
	NAM-CR	0.676(0.028)	0.664(0.024)	0.605(0.041)	0.697(0.029)
	Linear-SG.IE	0.674(0.024)	0.682(0.022)	0.653(0.038)	0.728(0.025)
	Linear-IA.IE	0.640(0.028)	0.650(0.023)	0.580(0.057)	0.680(0.036)
	DeepGE.IE	0.656(0.026)	0.674(0.018)	0.642(0.030)	0.713(0.030)
	Dense.IE	0.691(0.009)	0.654(0.010)	0.665(0.010)	0.694(0.011)
(b.IV) Nonlinear structure	Proposed	0.879(0.004)	0.861(0.013)	0.844(0.013)	0.883(0.005)
	NAM-SG.IE	0.871(0.008)	0.835(0.017)	0.832(0.028)	0.879(0.011)
	NAM-MCP.IE	0.850(0.009)	0.829(0.015)	0.814(0.021)	0.859(0.014)
	NAM-CR	0.871(0.009)	0.831(0.019)	0.831(0.025)	0.879(0.011)
	Linear-SG.IE	0.796(0.009)	0.826(0.011)	0.838(0.010)	0.885(0.007)
	Linear-IA.IE	0.793(0.010)	0.797(0.024)	0.821(0.025)	0.883(0.009)
	DeepGE.IE	0.755(0.016)	0.792(0.012)	0.773(0.022)	0.837(0.015)
	Dense.IE	0.703(0.008)	0.669(0.009)	0.685(0.007)	0.694(0.007)

Table S11: Computation time (in seconds) for different methods. $n = 500$. The computation time is measured as the average runtime during the grid search over candidate tuning parameter combinations. In each cell, mean (sd) based on 100 replicates.

Relationship between G and I variables	Method	Example 1	Example 2	Example 3	Example 4
(b.I) Banded structure	Proposed	122.3(31.0)	130.7(31.4)	101.1(10.1)	104.2(21.1)
	NAM-SG	40.3(2.9)	31.7(2.7)	42.4(22.3)	53.1(9.9)
	NAM-MCP	37.0(4.5)	29.3(2.5)	35.0(18.8)	41.9(7.9)
	NAM-CR	106.7(12.0)	80.4(9.2)	108.2(60.1)	129.3(27.6)
	AM-Bspline	13.8(6.1)	16.5(6.1)	12.8(3.3)	12.8(4.9)
	Linear-SG	19.7(9.9)	22.3(7.7)	20.8(4.2)	36.5(16.6)
	Linear-IA	13.6(7.5)	18.0(6.5)	14.1(2.9)	19.2(9.5)
	DeepGE	14.5(3.9)	11.6(3.4)	15.1(10.8)	20.0(7.8)
	Dense	10.3(2.6)	8.3(2.1)	9.7(5.1)	12.0(3.4)
	MA	60.1(33.1)	74.8(30.6)	59.1(16.3)	56.7(27.1)
(b.II) Diagonal structure	Proposed	121.9(34.1)	107.2(11.0)	89.7(9.6)	166.2(35.8)
	NAM-SG	53.3(7.7)	40.4(12.9)	39.0(9.8)	48.6(12.8)
	NAM-MCP	45.0(6.1)	35.6(11.4)	33.8(8.3)	42.5(8.7)
	NAM-CR	152.2(30.1)	109.9(38.0)	100.3(21.1)	130.6(33.7)
	AM-Bspline	12.9(5.8)	11.5(2.1)	10.8(2.3)	22.8(7.6)
	Linear-SG	19.1(8.6)	18.2(3.2)	16.1(2.6)	21.1(1.6)
	Linear-IA	14.6(7.8)	13.1(2.1)	12.8(2.3)	13.0(2.0)
	DeepGE	21.9(8.8)	13.2(6.3)	12.8(5.1)	17.7(8.2)
	Dense	12.6(2.6)	10.0(4.0)	9.7(3.5)	11.6(2.8)
	MA	54.0(30.1)	57.3(11.3)	43.8(12.0)	116.3(43.5)
(b.III) Randomly-set structure	Proposed	99.9(10.1)	109.7(12.7)	95.1(25.7)	164.8(43.1)
	NAM-SG	49.4(12.4)	36.6(3.0)	35.6(4.5)	64.4(23.7)
	NAM-MCP	41.9(8.0)	36.9(4.1)	30.3(4.4)	46.2(15.0)
	NAM-CR	128.3(29.8)	95.3(7.8)	96.8(14.5)	157.2(58.7)
	AM-Bspline	10.6(2.0)	12.1(2.9)	11.5(4.5)	20.5(7.4)
	Linear-SG	15.2(2.1)	19.6(4.6)	17.2(6.7)	28.4(14.8)
	Linear-IA	11.2(2.1)	15.2(5.0)	11.7(4.1)	15.5(9.4)
	DeepGE	19.2(7.6)	11.8(2.9)	13.2(4.6)	23.2(12.8)
	Dense	12.5(4.2)	9.9(2.6)	9.0(2.3)	15.1(5.4)
	MA	47.3(12.9)	56.2(15.2)	51.5(24.2)	102.9(42.0)
(b.IV) Nonlinear structure	Proposed	96.0(6.3)	104.9(20.6)	91.8(22.1)	152.4(33.5)
	NAM-SG	51.6(11.4)	38.5(6.2)	41.0(13.5)	55.2(8.2)
	NAM-MCP	41.2(8.8)	33.2(4.1)	34.2(10.4)	45.1(7.8)
	NAM-CR	135.7(24.5)	103.6(21.0)	99.0(26.9)	142.9(24.8)
	AM-Bspline	10.5(2.1)	14.2(6.1)	9.7(2.5)	13.8(3.3)
	Linear-SG	16.8(3.1)	22.3(9.6)	15.8(4.4)	28.6(15.8)
	Linear-IA	10.9(1.9)	13.9(5.8)	9.8(2.7)	14.6(9.8)
	DeepGE	20.5(6.8)	13.8(4.7)	13.2(6.9)	19.3(6.3)
	Dense	12.0(3.5)	9.5(2.8)	10.1(4.9)	12.8(3.1)
	MA	51.3(11.3)	67.0(37.8)	40.3(13.5)	74.8(20.1)

Table S12: Computation time (in seconds) for different methods. $n = 1000$. The computation time is measured as the average runtime during the grid search over candidate tuning parameter combinations. In each cell, mean (sd) based on 100 replicates.

Relationship between G and I variables	Method	Example 1	Example 2	Example 3	Example 4
(b.I) Banded structure	Proposed	188.1(50.5)	138.0(15.9)	119.3(11.1)	165.5(68.7)
	NAM-SG	73.3(17.1)	58.7(17.7)	64.4(21.3)	56.2(3.5)
	NAM-MCP	73.2(14.2)	63.3(19.7)	65.1(23.1)	57.8(4.2)
	NAM-CR	185.0(40.2)	140.8(47.3)	148.2(48.9)	146.5(13.2)
	AM-Bspline	14.9(6.6)	15.1(2.7)	11.0(2.0)	15.6(9.1)
	Linear-SG	16.8(7.8)	17.1(2.6)	14.1(2.8)	18.5(1.6)
	Linear-IA	11.4(5.9)	13.1(2.8)	10.1(1.9)	11.9(1.9)
	DeepGE	17.4(10.6)	13.9(6.5)	15.7(8.6)	15.7(4.1)
	Dense	21.6(5.2)	17.5(5.8)	19.3(8.4)	16.3(2.7)
	MA	135.3(68.5)	119.3(30.5)	88.6(16.3)	145.2(90.0)
(b.II) Diagonal structure	Proposed	151.1(18.8)	149.1(21.3)	161.2(34.8)	149.8(38.7)
	NAM-SG	49.9(5.1)	58.8(24.3)	56.9(7.8)	81.4(26.2)
	NAM-MCP	54.1(5.8)	61.7(22.0)	59.1(7.4)	81.9(25.0)
	NAM-CR	141.5(17.4)	150.7(58.4)	144.1(25.5)	204.3(69.3)
	AM-Bspline	13.7(2.9)	13.2(2.7)	15.1(6.8)	13.6(6.6)
	Linear-SG	17.2(3.7)	17.4(3.5)	17.7(6.9)	18.8(1.9)
	Linear-IA	12.4(3.1)	12.1(2.9)	12.3(5.0)	11.7(1.9)
	DeepGE	13.6(5.4)	15.4(9.4)	13.8(5.1)	18.0(10.4)
	Dense	15.5(3.1)	16.5(7.4)	17.0(3.7)	23.9(8.8)
	MA	129.5(32.0)	121.5(25.3)	127.7(57.3)	138.5(72.3)
(b.III) Randomly-set structure	Proposed	138.2(11.2)	144.6(21.0)	132.5(37.1)	160.2(65.8)
	NAM-SG	70.7(15.2)	73.8(9.4)	62.0(13.2)	68.3(12.9)
	NAM-MCP	68.8(14.2)	76.8(11.6)	61.6(10.1)	69.0(14.7)
	NAM-CR	167.2(39.8)	178.6(32.7)	144.1(24.9)	166.2(37.8)
	AM-Bspline	12.4(2.6)	13.4(3.8)	14.0(5.8)	14.1(9.9)
	Linear-SG	14.4(3.3)	17.3(5.0)	17.0(8.1)	19.1(1.6)
	Linear-IA	11.0(2.4)	12.6(3.0)	12.0(6.3)	11.5(2.0)
	DeepGE	17.1(6.6)	16.6(5.9)	14.8(5.2)	15.8(6.8)
	Dense	20.9(5.6)	22.1(5.1)	18.2(4.7)	20.5(5.0)
	MA	109.1(22.6)	118.9(34.5)	122.7(52.9)	133.1(92.8)
(b.IV) Nonlinear structure	Proposed	167.3(27.4)	173.9(46.4)	177.4(61.1)	480.6(25.1)
	NAM-SG	72.8(11.3)	57.5(16.0)	59.6(11.9)	77.5(12.0)
	NAM-MCP	72.4(14.3)	58.9(14.4)	61.6(11.8)	78.1(12.8)
	NAM-CR	175.1(35.4)	131.8(36.1)	140.6(27.8)	192.1(39.0)
	AM-Bspline	15.7(5.3)	17.5(6.4)	14.9(8.4)	23.0(3.7)
	Linear-SG	18.5(6.5)	20.2(7.4)	20.4(12.5)	20.6(9.5)
	Linear-IA	12.1(4.1)	13.9(5.7)	13.1(7.9)	10.3(5.4)
	DeepGE	22.8(9.3)	12.3(5.5)	15.2(7.7)	22.8(9.7)
	Dense	21.5(4.9)	17.1(5.1)	17.9(4.9)	22.6(5.5)
	MA	141.8(45.5)	169.6(77.0)	144.5(83.1)	234.4(31.3)

Table S13: Simulation results when the "main effects, interactions" hierarchy is partially violated. We assume there are 15 main G effects and 25 interaction effects among which the corresponding main G effects for 5 interaction terms are not significant. The relationship between G and I variables has a the nonlinear structure (b.IV). $n = 500$. In each cell, mean (sd) based on 100 replicates.

	cutoff	Main effects (M)		Interactions (I)		C-index
		TP	FP	TP	FP	
Example 1	Proposed	14.1(0.9)	3.1(3.9)	21.5(1.9)	6.3(7.4)	0.877(0.016)
	NAM-SG	12.7(2.2)	4.3(4.1)	18.4(4.5)	5.9(5.0)	0.845(0.034)
	NAM-MCP	10.3(2.4)	2.0(3.0)	11.6(3.1)	10.7(10.6)	0.790(0.026)
	NAM-CR	13.5(1.0)	2.2(1.7)	19.3(2.7)	4.4(3.2)	0.856(0.020)
	AM-Bspline	12.8(1.5)	17.3(6.4)	12.2(2.5)	30.5(8.1)	0.734(0.016)
	Linear-SG	11.4(1.5)	2.7(5.0)	14.4(3.2)	4.6(7.7)	0.762(0.022)
	Linear-IA	11.6(1.4)	1.5(1.3)	14.8(2.5)	2.1(2.1)	0.767(0.019)
	DeepGE	4.2(2.6)	8.6(6.9)	5.1(4.0)	13.4(12.4)	0.650(0.053)
	Dense	-	-	-	-	0.631(0.007)
	MA	13.8(1.0)	3.4(1.8)	12.1(2.6)	25.2(6.6)	-
Example 2	Proposed	11.0(1.9)	1.7(3.1)	10.8(3.2)	4.4(3.8)	0.780(0.023)
	NAM-SG	8.7(2.9)	2.7(3.7)	7.8(4.2)	4.9(6.3)	0.744(0.033)
	NAM-MCP	8.0(2.7)	1.3(1.7)	4.2(2.4)	6.9(7.7)	0.725(0.031)
	NAM-CR	10.9(2.4)	3.2(3.5)	9.2(3.2)	6.0(5.5)	0.763(0.027)
	AM-Bspline	13.5(2.1)	22.3(8.9)	8.1(3.0)	29.0(10.7)	0.717(0.024)
	Linear-SG	8.2(1.9)	1.8(2.6)	6.5(2.3)	3.5(4.1)	0.739(0.023)
	Linear-IA	8.4(1.9)	0.5(0.8)	6.1(2.6)	2.1(1.4)	0.745(0.020)
	DeepGE	4.8(2.5)	8.9(6.5)	3.8(2.2)	15.1(11.1)	0.666(0.045)
	Dense	-	-	-	-	0.595(0.008)
	MA	14.6(0.7)	3.1(2.0)	5.9(2.2)	20.7(5.8)	-
Example 3	Proposed	11.1(2.0)	3.0(3.4)	18.4(3.2)	5.0(5.0)	0.788(0.031)
	NAM-SG	8.3(2.5)	3.0(4.0)	12.2(4.6)	4.5(5.7)	0.730(0.036)
	NAM-MCP	4.1(2.7)	1.4(2.3)	7.7(4.1)	8.7(8.7)	0.666(0.047)
	NAM-CR	10.0(2.1)	3.4(2.5)	15.6(3.5)	5.3(4.2)	0.759(0.033)
	AM-Bspline	8.7(2.2)	20.9(5.8)	14.5(2.6)	30.6(7.6)	0.666(0.020)
	Linear-SG	8.3(2.0)	3.4(4.3)	12.4(3.5)	6.0(7.0)	0.727(0.036)
	Linear-IA	8.4(1.9)	1.4(1.1)	12.8(3.6)	2.6(1.9)	0.738(0.030)
	DeepGE	3.6(2.2)	8.1(5.5)	4.6(3.2)	13.7(10.0)	0.606(0.057)
	Dense	-	-	-	-	0.606(0.006)
	MA	10.9(1.7)	2.6(1.7)	15.5(2.2)	18.3(4.2)	-
Example 4	Proposed	13.5(1.2)	2.8(2.7)	20.4(2.7)	5.1(3.5)	0.874(0.022)
	NAM-SG	12.9(2.1)	3.9(3.1)	17.7(5.1)	5.7(4.4)	0.852(0.043)
	NAM-MCP	10.0(3.0)	1.5(1.9)	10.4(4.2)	10.8(9.2)	0.787(0.038)
	NAM-CR	12.9(2.0)	3.2(2.6)	18.4(3.6)	5.8(5.0)	0.857(0.036)
	AM-Bspline	12.5(2.0)	21.6(7.2)	10.0(2.7)	30.0(8.9)	0.708(0.020)
	Linear-SG	13.9(1.1)	3.4(3.2)	21.2(2.9)	4.9(4.7)	0.888(0.034)
	Linear-IA	14.4(0.8)	2.6(1.0)	22.3(2.2)	2.8(2.0)	0.903(0.022)
	DeepGE	5.9(3.1)	6.1(7.0)	6.9(3.7)	10.8(13.0)	0.712(0.067)
	Dense	-	-	-	-	0.613(0.006)
	MA	14.0(1.0)	3.4(1.8)	13.2(2.2)	24.0(5.7)	-

Table S14: Simulation results when the "main effects, interactions" hierarchy is partially violated. We assume there are 15 main G effects and 25 interaction effects among which the corresponding main G effects for 5 interaction terms are not significant. The relationship between G and I variables has a the nonlinear structure (b.IV). $n = 1000$. In each cell, mean (sd) based on 100 replicates.

	cutoff	Main effects (M)		Interactions (I)		C-index
		TP	FP	TP	FP	
Example 1	Proposed	15.0(0.0)	4.0(1.0)	24.5(0.7)	7.4(4.1)	0.918(0.007)
	NAM-SG	14.9(0.3)	4.9(2.3)	24.1(1.1)	6.1(4.8)	0.909(0.012)
	NAM-MCP	14.3(1.0)	0.1(0.5)	20.1(2.8)	2.1(2.0)	0.882(0.023)
	NAM-CR	14.8(0.5)	2.8(0.9)	23.2(1.1)	1.0(1.1)	0.904(0.011)
	AM-Bspline	14.7(0.7)	13.7(9.3)	18.1(2.9)	15.7(6.8)	0.813(0.014)
	Linear-SG	14.5(1.1)	3.1(1.6)	22.0(2.8)	5.0(3.4)	0.815(0.017)
	Linear-IA	13.5(1.4)	1.5(1.0)	19.4(2.6)	1.0(1.0)	0.799(0.016)
	DeepGE	4.7(4.0)	6.4(5.4)	6.1(5.6)	10.2(11.8)	0.666(0.089)
	Dense	-	-	-	-	0.655(0.006)
	MA	15.0(0.2)	3.8(2.0)	19.7(2.3)	22.3(6.3)	-
Example 2	Proposed	14.6(0.7)	2.9(1.4)	21.4(2.5)	8.8(3.5)	0.861(0.021)
	NAM-SG	12.9(2.4)	3.1(3.7)	14.7(4.9)	5.0(7.0)	0.804(0.032)
	NAM-MCP	12.5(2.4)	0.5(1.1)	10.0(3.7)	4.3(5.4)	0.797(0.028)
	NAM-CR	11.7(1.8)	0.6(1.2)	11.2(3.5)	0.9(1.6)	0.790(0.018)
	AM-Bspline	15.0(0.1)	16.9(7.1)	15.5(4.1)	11.8(6.7)	0.804(0.018)
	Linear-SG	12.3(3.2)	2.6(2.8)	13.0(5.3)	7.3(5.0)	0.784(0.032)
	Linear-IA	7.9(1.8)	0.1(0.2)	5.6(2.6)	0.6(0.9)	0.746(0.022)
	DeepGE	4.5(3.2)	4.7(3.6)	3.8(3.5)	8.8(6.4)	0.674(0.065)
	Dense	-	-	-	-	0.611(0.006)
	MA	15.0(0.0)	3.6(2.1)	11.0(2.3)	18.9(4.7)	-
Example 3	Proposed	14.3(0.9)	4.1(1.2)	24.0(1.2)	7.5(3.4)	0.859(0.015)
	NAM-SG	13.5(1.4)	5.0(4.3)	22.5(2.4)	4.9(6.5)	0.831(0.021)
	NAM-MCP	9.3(2.0)	0.2(0.5)	18.5(3.6)	3.0(4.9)	0.795(0.037)
	NAM-CR	12.4(1.5)	2.4(1.1)	20.9(2.5)	0.9(0.8)	0.818(0.021)
	AM-Bspline	13.2(1.7)	15.5(7.5)	21.6(2.1)	13.1(6.6)	0.778(0.019)
	Linear-SG	13.1(2.0)	4.4(2.3)	22.0(3.4)	7.8(4.3)	0.817(0.029)
	Linear-IA	9.9(2.0)	1.1(0.9)	16.6(3.8)	0.8(1.0)	0.778(0.030)
	DeepGE	4.8(3.2)	7.8(8.2)	7.2(5.3)	12.8(13.6)	0.647(0.081)
	Dense	-	-	-	-	0.624(0.007)
	MA	14.4(0.8)	3.3(1.7)	22.1(1.7)	17.5(4.1)	-
Example 4	Proposed	14.9(0.3)	3.7(1.1)	24.0(1.4)	7.0(4.3)	0.921(0.014)
	NAM-SG	14.8(0.7)	4.5(2.1)	23.8(1.7)	5.3(5.4)	0.919(0.019)
	NAM-MCP	14.5(0.8)	0.1(0.4)	19.4(2.6)	2.6(3.9)	0.887(0.024)
	NAM-CR	14.6(0.6)	2.6(1.2)	22.7(1.8)	1.1(1.1)	0.910(0.017)
	AM-Bspline	14.6(0.6)	14.2(8.3)	15.8(2.5)	11.6(4.9)	0.800(0.015)
	Linear-SG	14.9(0.6)	3.7(1.0)	24.0(1.6)	2.9(2.3)	0.927(0.016)
	Linear-IA	14.9(0.4)	3.0(1.0)	23.5(1.2)	0.7(1.0)	0.921(0.012)
	DeepGE	5.1(4.1)	2.7(4.1)	6.0(5.1)	4.3(6.5)	0.699(0.106)
	Dense	-	-	-	-	0.638(0.007)
	MA	14.9(0.2)	4.0(2.0)	20.3(2.2)	23.0(5.1)	-

Table S15: Robustness evaluation for varying the cutoff c^{corr}. We set c^{corr} as different quantiles of $|c^{\text{corr}}_{j,j'}|$'s for evaluation. The relationship between G and I variables has a the randomly-set structure (b.II). $n = 500$. In each cell, mean (sd) based on 100 replicates.

	Quantile for c^{corr}	Main effects (M)		Interactions (I)		C-index
		TP	FP	TP	FP	
Example 1	0.95	14.3(0.9)	0.2(0.9)	24.1(1.3)	3.7(2.3)	0.899(0.013)
	0.9	14.4(0.7)	0.3(0.8)	24.0(1.3)	3.0(2.4)	0.901(0.015)
	0.6	14.3(0.8)	0.9(3.7)	23.9(1.5)	4.0(5.7)	0.898(0.016)
Example 2	0.95	11.9(1.9)	0.5(0.7)	15.2(4.1)	3.1(2.3)	0.801(0.029)
	0.9	12.0(1.9)	1.0(2.8)	15.4(3.5)	4.1(5.8)	0.803(0.026)
	0.6	11.9(2.2)	1.0(2.9)	15.1(4.3)	4.3(6.0)	0.802(0.030)
Example 3	0.95	12.2(1.6)	0.6(1.1)	21.1(2.8)	3.4(2.4)	0.818(0.026)
	0.9	12.7(1.4)	1.4(2.6)	21.9(2.3)	4.7(4.4)	0.823(0.022)
	0.6	12.5(1.6)	1.1(2.1)	21.6(2.8)	4.6(3.6)	0.821(0.026)
Example 4	0.95	13.9(1.3)	1.1(3.9)	23.3(2.4)	4.0(6.7)	0.899(0.025)
	0.9	14.0(1.2)	0.2(0.7)	23.2(2.4)	2.8(2.7)	0.900(0.025)
	0.6	13.9(1.2)	0.8(2.6)	23.1(2.5)	3.5(4.1)	0.899(0.024)

Table S16: Robustness evaluation for varying the cutoff c^{corr}. We set c^{corr} as different quantiles of $|c^{\text{corr}}_{j,j'}|$'s for evaluation. The relationship between G and I variables has a randomly-set structure (b.II). $n = 1000$. In each cell, mean (sd) based on 100 replicates.

	Quantile for c^{corr}	Main effects (M)		Interactions (I)		C-index
		TP	FP	TP	FP	
Example 1	0.95	15.0(0.0)	0.0(0.0)	25.0(0.0)	2.9(2.7)	0.922(0.002)
	0.9	15.0(0.0)	0.0(0.0)	25.0(0.0)	2.7(2.4)	0.922(0.002)
	0.6	15.0(0.0)	0.0(0.0)	25.0(0.1)	2.1(2.0)	0.922(0.003)
Example 2	0.95	14.7(0.5)	0.8(1.2)	24.0(1.4)	7.3(3.0)	0.877(0.018)
	0.9	14.8(0.4)	0.7(1.1)	24.0(1.6)	7.5(3.2)	0.874(0.017)
	0.6	14.7(0.6)	0.7(1.4)	23.9(1.6)	7.5(3.6)	0.876(0.019)
Example 3	0.95	14.5(0.8)	0.3(0.8)	24.8(0.8)	3.8(2.9)	0.870(0.011)
	0.9	14.5(0.7)	0.2(0.5)	24.8(0.8)	4.2(2.9)	0.871(0.010)
	0.6	14.6(0.8)	0.3(0.7)	24.8(0.7)	4.7(2.8)	0.871(0.012)
Example 4	0.95	14.9(0.3)	0.0(0.2)	24.9(0.5)	2.1(1.8)	0.928(0.007)
	0.9	14.9(0.3)	0.0(0.0)	24.9(0.6)	2.3(2.0)	0.928(0.007)
	0.6	14.9(0.3)	0.0(0.0)	24.9(0.5)	2.1(1.7)	0.928(0.007)

S3 Additional results in real data analysis

S3.1 Lung Cancer

- Definition for RV coefficients The RV-coefficient (Smilde et al., 2009) is a matrix correlation measure, characterizing the connection between two sets of variables, and is a generalization of Pearson correlation. For $n \times r$ matrix $\mathbf{X}$ and $n \times p$ matrix $\mathbf{Y}$, the RV-coefficient is defined as:

$$r(\mathbf{X}, \mathbf{Y}) = \frac{tr\left(\mathbf{X}\mathbf{X}^\top \mathbf{Y}\mathbf{Y}^\top\right)}{\sqrt{tr\left(\mathbf{X}\mathbf{X}^\top\right)^2 tr\left(\mathbf{Y}\mathbf{Y}^\top\right)^2}}.$$

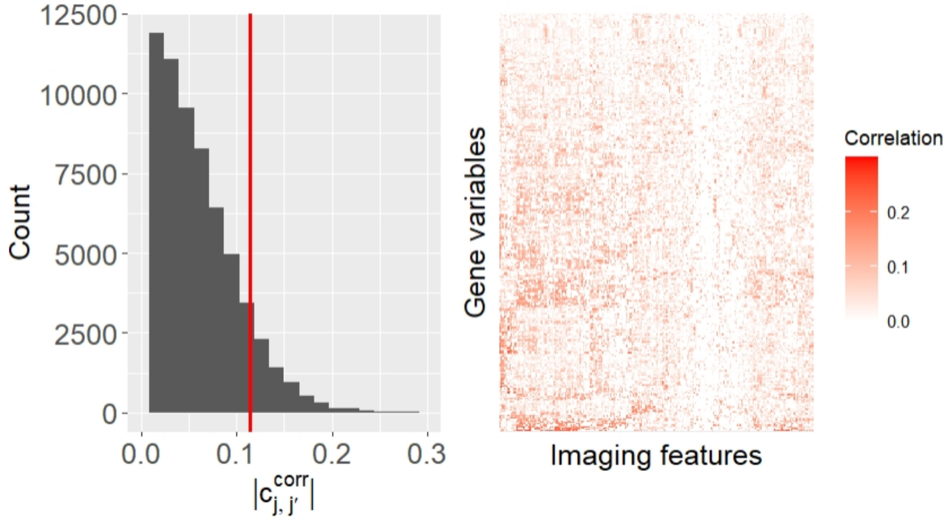

Figure S1: Left: Histogram for the absolute Spearman correlation coefficients ($c^{\text{corr}}_{j,j'}$'s) matrix for gene variables and imaging features in lung cancer dataset. The red line represents the cutoff c^{corr}. Right: Heatmap for corresponding parameters $c_{j,j'}$'s with the cutoff being the 90% quantile of the absolute values of the correlation coefficients.

Table S17: Lung cancer data analysis: identification results for imaging data-based interaction interaction model. The asterisks in the table indicate selected variables.

Main I	Interactions			
	Age	Sex	Smoking	Stage
Texture_Entropy_maskosingray_3_01_256	*		*	*
Texture_Entropy_maskosingray_3_03_256		*	*	*
AreaShape_BoundingBoxMaximum_X		*		*
AreaShape_ConvexArea	*			
AreaShape_Orientation	*	*	*	*
AreaShape_Perimeter	*	*	*	
AreaShape_Zernike_3_3	*		*	*
AreaShape_Zernike_4_4	*			
Location_Center_Y.2	*	*		*
Texture_Contrast_ImageAfterMath_3_02_256				*
Texture_DifferenceVariance_ImageAfterMath_3_03_256		*	*	
Granularity_15_ImageAfterMath.1			*	*
Threshold_OrigThreshold_Identifyhemasub2				*
Threshold_SumOfEntropies_Identifyhemasub2		*		*
Threshold_SumOfEntropies_identifyhemaprimarynuclei	*	*		*
Threshold_SumOfEntropies_identifytissueregion	*	*	*	*

Table S18: Lung cancer data analysis using the proposed and alternative methods. The “Num” columns show the number of variables identified by methods, the “Overlap Num” columns display the count of variables identified by both the proposed and alternative methods, and the “RV” columns contain the RV coefficients for these overlapping variables.

Method	Main G effects			Interaction effects		
	Num	Num overlap	RV	Num inter	Num overlap	RV
Proposed	12	-	-	33	-	-
NAM-SG	11	4	0.48	14	5	0.26
NAM-MCP	4	3	0.47	11	2	0.23
NAM-CR	8	3	0.40	11	4	0.24
AM-Bspline	10	4	0.48	14	5	0.29
Linear-SG	7	4	0.50	11	6	0.32
Linear-IA	7	4	0.50	11	6	0.32
DeepGE	8	3	0.36	15	4	0.25
MA	9	2	0.36	16	1	0.09

S3.2 Skin Cancer

- **The literature search results for the identification findings**

  The expression level of gene *CAV3* is positively correlated with autophagy biomarkers associated with melanoma progression, prognosis, and the development of resistance to BRAFi, potentially contributing to novel therapeutic strategies. Gene *FCGR2A* is strongly linked to overall survival, oncogene mutations, tumor stemness, immune infiltration, treatment responses, and various biological processes in SKCM patients. Gene *NLRC5* is expressed in both immune and melanoma cells, and its expression is regulated by SPI1 and DNA methylation. Gene *NLRC5* is significantly associated with Breslow thickness, Clark level, recurrence, pathologic T stage, and ulceration status in melanoma. Phosphorylation of gene *PKP1* regulates epidermal differentiation and skin tumorigenesis. This gene is critical to the immune microenvironment and plays a key role in melanoma cell proliferation, migration, invasion, and the cell cycle. Gene *SCG2* is involved in the immune pathway and serves as a prognostic marker in SKCM, influencing tumor immunity through the regulation of immune cell infiltration and monocyte polarization. In the imaging-based interaction model, the proposed method identifies 5 I main effects, 5 E main effects, and 21 interaction effects, with the detailed results presented in Supplementary Table S12.

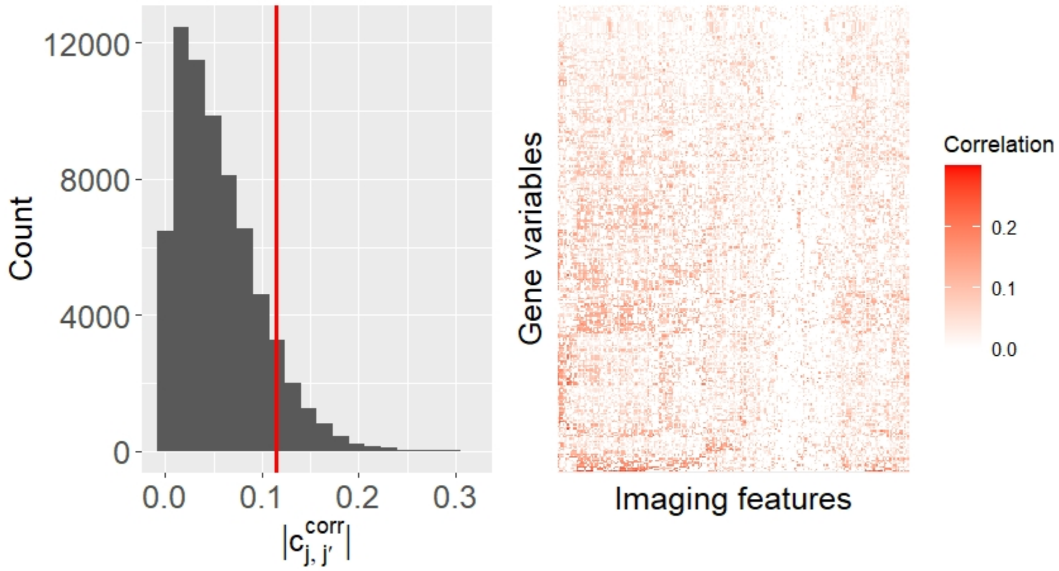

Figure S2: Left: Histogram for the absolute Spearman correlation coefficients ($c^{\text{corr}}_{j,j'}$'s) matrix for gene variables and imaging features in skin cancer dataset. The red line represents the cutoff c^{corr}. Right: Heatmap for corresponding parameters $c_{j,j'}$'s with the cutoff being the 90% quantile of the absolute values of the correlation coefficients.

Table S19: Skin cancer data analysis: identification results for imaging based interaction interaction model. The asterisks in the table indicate selected variables.

Main G	Interactions				
	Age	Sex	Breslow	Clark	Stage
Location_Center_X	*	*			*
Location_Center_X.1	*		*	*	*
Granularity_16_ImageAfterMath	*	*	*		*
AreaOccupied_AreaOccupied_Identifyeosinprimarycytoplasm	*	*	*	*	*
Granularity_4_ImageAfterMath.1	*	*	*	*	*

Table S20: Skin cancer data analysis using the proposed and alternative methods. The "Num" columns show the number of variables identified by methods, the "Overlap Num" columns display the count of variables identified by both the proposed and alternative methods, and the "RV" columns contain the RV coefficients for these overlapping variables.

Method	Main G effects			Interaction effects		
	Num	Num overlap	RV	Num	Num overlap	RV
Proposed	10	-	-	19	-	-
NAM-SG	16	7	0.60	19	10	0.53
NAM-MCP	3	2	0.41	8	4	0.41
NAM-CR	10	4	0.48	14	6	0.43
AM-Bspline	12	4	0.46	11	5	0.42
Linear-SG	8	5	0.57	14	8	0.47
Linear-IA	8	5	0.57	14	8	0.47
DeepGE	15	1	0.31	26	1	0.40
MA	1	1	0.04	7	3	0.29

S4 Extentions

We may consider the following including but not limited to potential extentions:

1. The proposed method neural additive model (NAM) structure for G–E analysis and the penalized estimation method can be potentially extended to broader interaction analysis applications, including gene-gene interaction analysis (Cui et al., 2022), higher order interaction analysis (Balasubramanian, 2021), and functional interaction model (Liang et al., 2023).

2. We can extend the assisted learning strategy for G–E modeling when we additionally have both other omics data and image data. Furthermore, considering the regulatory relationship between different omics data (Woo et al., 2020; Li et al., 2023), we can also consider modifying the proposed assisted learning to incorporate such "directional" information.

3. Bayesian neural network (BNN) (Blundell et al., 2015) has many advantages, especially on quantifying uncertainty. Thus, based on the proposed neural network architecture for G–E interaction, it has the potential to extend to BNN-based interaction model.

References

Balasubramanian, K. (2021), “Nonparametric Modeling of Higher-Order Interactions via Hypergraphons,” *Journal of Machine Learning Research*, 22, 1–35.

Blundell, C., Cornebise, J., Kavukcuoglu, K., and Wierstra, D. (2015), “Weight Uncertainty in Neural Networks,” in *Proceedings of the 32nd International Conference on International Conference on Machine Learning - Volume 37*, Lille, France: JMLR.org, ICML’15, pp. 1613–1622.

Cui, T., El Mekkaoui, K., Reinvall, J., Havulinna, A. S., Marttinen, P., and Kaski, S. (2022), “Gene-Gene Interaction Detection with Deep Learning,” *Communications Biology*, 5, 1238.

Fang, K., Li, J., Xu, Y., Ma, S., and Zhang, Q. (2023), “Gene–Environment Interaction Analysis under the Cox Model,” *Annals of the Institute of Statistical Mathematics*, 75, 931–948.

Li, Z., Gao, E., Zhou, J., Han, W., Xu, X., and Gao, X. (2023), “Applications of Deep Learning in Understanding Gene Regulation,” *Cell Reports Methods*, 3, 100384.

Liang, W., Zhang, Q., and Ma, S. (2023), “Locally Sparse Quantile Estimation for a Partially Functional Interaction Model,” *Computational Statistics & Data Analysis*, 186, 107782.

Smilde, A. K., Kiers, H. A. L., Bijlsma, S., Rubingh, C. M., and van Erk, M. J. (2009), “Matrix Correlations for High-Dimensional Data: The Modified RV-coefficient,” *Bioinformatics*, 25, 401–405.

Woo, G., Fernandez, M., Hsing, M., Lack, N. A., Cavga, A. D., and Cherkasov, A. (2020), “DeepCOP: Deep Learning-Based Approach to Predict Gene Regulating Effects of Small Molecules,” *Bioinformatics*, 36, 813–818.
